# Synthesis and Investigation of Electro-Optical Properties of H-Shape Dibenzofulvene Derivatives

**DOI:** 10.3390/molecules27031091

**Published:** 2022-02-06

**Authors:** Maria Michela Giangregorio, Salvatore Gambino, Eduardo Fabiano, Mauro Leoncini, Antonio Cardone, Giuseppina Anna Corrente, Amerigo Beneduci, Gianluca Accorsi, Giuseppe Gigli, Maria Losurdo, Roberto Termine, Agostina-Lina Capodilupo

**Affiliations:** 1 Institute of Nanotechnology (CNR-NANOTEC), c/o Department of Chemistry, University of Bari, Via Orabona 4, 70126 Bari, Italy; michelaria.giangregorio@nanotec.cnr.it (M.M.G.); maria.losurdo@cnr.it (M.L.); 2 Institute of Nanotechnology (CNR-NANOTEC), c/o Campus Ecotekne, University of Salento, Via Monteroni, 73100 Lecce, Italy; salvatore.gambino@nanotec.cnr.it (S.G.); mauro.leoncini@nanotec.cnr.it (M.L.); gianluca.accorsi@nanotec.cnr.it (G.A.); giuseppe.gigli@nanotec.cnr.it (G.G.); 3 Institute for Microelectronics and Microsystems (CNR-IMM), c/o Campus Ecotekne, University of Salento, Via Monteroni, 73100 Lecce, Italy; eduardo.fabiano@cnr.it; 4 Centre for Biomolecular Nanotechnologies @UNILE, Istituto Italiano di Tecnologia (IIT), 73010 Lecce, Italy; 5 Department of Mathematics and Physics “Ennio de Giorgi”, University of Salento, 73100 Lecce, Italy; 6 Institute of Chemistry of OrganoMetallic Compounds, ICCOM, Italian National Council of Research, CNR, Via Orabona 4, 70125 Bary, Italy; cardone@ba.iccom.cnr.it; 7 Department of Chemistry and Chemical Technologies, University of Calabria, Via P. Bucci, Cubo 15D, 87036 Arcavacata di Rende, Italy; giuseppina.corrente@unical.it (G.A.C.); amerigo.beneduci@unical.it (A.B.); 8 Institute of Nanotechnology (CNR-NANOTEC), c/o University of Calabria, Via P. Bucci, 87036 Rende, Italy; roberto.termine@cnr.it

**Keywords:** dibenzofulvene, arylamine, ellipsometry spectroscopy, Raman spectroscopy

## Abstract

We have synthetized two classes of dibenzofulvene-arylamino derivatives with an H-shape design, for a total of six different molecules. The molecular structures consist of two D-A-D units connected by a thiophene or bitiophene bridge, using diarylamino substituents as donor groups anchored to the 2,7- (Group A) and 3,6- (Group B) positions of the dibenzofulvene backbone. The donor units and the thiophene or bithiophene bridges were used as chemico-structural tools to modulate electro-optical and morphological-electrical properties. A combination of experiments, such as absorption measurements (UV-Vis spectroscopy), cyclic voltammetry, ellipsometry, Raman, atomic force microscopy, TD-DFT calculation and hole-mobility measurements, were carried out on the synthesized small organic molecules to investigate the differences between the two classes and therefore understand the relevance of the molecular design of the various properties. We found that the anchoring position on dibenzofulvene plays a crucial key for fine-tuning the optical, structural, and morphological properties of molecules. In particular, molecules with substituents in 2,7 positions (Group A) showed a lower structural disorder, a larger molecular planarity, and a lower roughness.

## 1. Introduction

**Figure 1:**
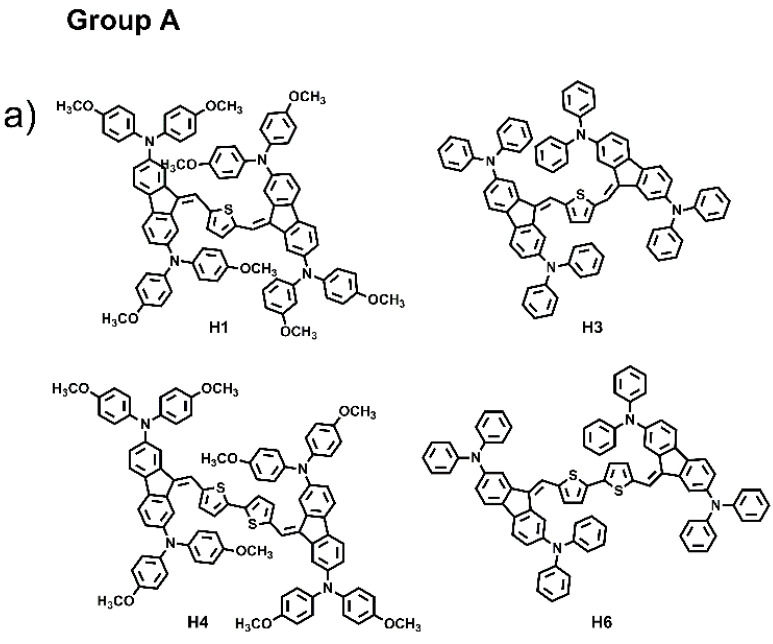

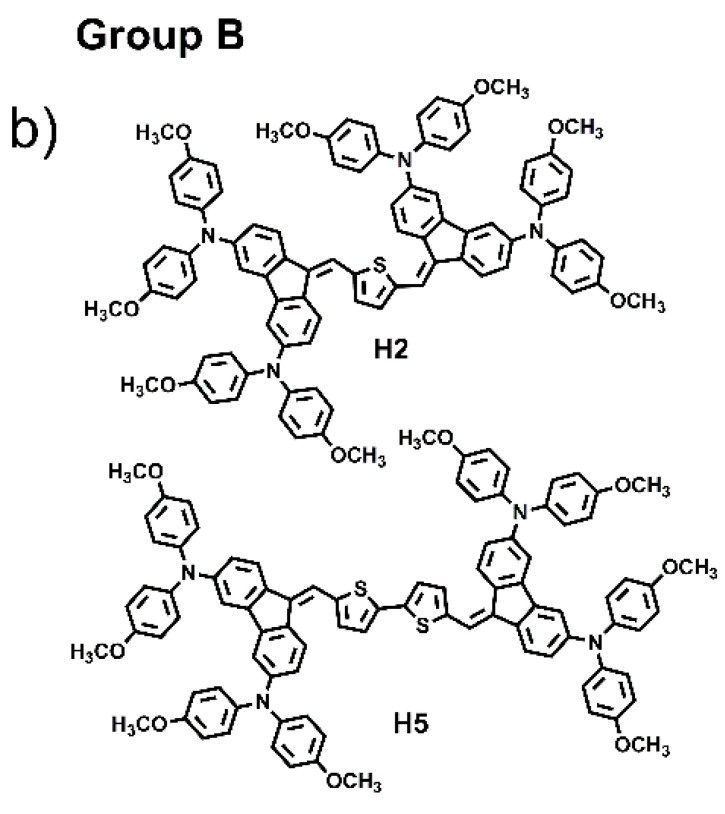
Chemical structures of **H1–H6**.

The combination of several organic structural motifs such as phenylene-vinylene, thiophene, pyrrole, triarylamines and fluorene derivates is widely studied because it gives life to a very important class of electroactive and photoactive compounds, used in several sector of optoelectronics, such as organic light-emitting diodes (OLED) [1], organic field-effect transistors (OFET) [2], electrochromic and electrofluorochromic devices (ECDs and EFCDs) [3,4,5], dye-sensitized solar cells (DSSCs) [6,7,8], as well as used as fluorescent probes in bioimaging applications [9,10,11]. In particular, in the last decade, great attention was paid to a large variety of organic molecule semiconductors with hole-transporting properties since they play a pivotal part for achieving, for example, high performances in perovskite solar cells (PSCs), ensuring efficient hole extraction and transportation, and the suppression of photogenerated carrier recombination [12,13,14]. Spirolink compounds, 3,4-ethylenedioxythiophene analogues and phthalocyanine derivatives, phenoxazine, phenothiazine, thiophene, anthracene and carbazole, are commonly used as core units to synthesize hole transport materials (HTMs). Their planar section favours strong π-π interaction leading to high hole mobility, and, at the same time, to aggregation phenomena and to the formation of films with excessively large crystalline domains, resulting in coarse surface morphology with low-quality hole-transporting layer thin film, which can be harmful for the improvement of cell performance. Thus, it is very important to design a new class of materials with molecular structures that have easy processability, are suitable to form good films, and that exhibit high charge mobility [15]. To probe the influences of molecular conformation on the electronic and charge transport properties of small molecules and to find more efficient central units, here we have designed and synthesized six small molecules, “H-shaped”, based on two units of dibenzofulvene (DBF) linked together by thiophene or bithiophene rings, and witharylaminomoieties used as electron donor groups anchored at the 2,7 and 3,6-DBF positions, as shown in Figure 1. The electronic and optical properties of all materials were investigated and combined experiments such as ellipsometry, Raman, atomic force microscopy, TD-DFT computation and hole-mobility investigations were carried out. Based on the position of the arylamino substituents linked to the DBF unit, the six molecules were divided in two groups: Group A, which includes the molecules labelled as **H1**, **H3**, **H4** and **H6**, characterized by the arylamino substituents linked to the 2,7-positions; Group B, which includes the molecules **H2**, and **H5**, characterized by the arylamino substituents linked to the 3,6-positions. **H1** and **H2** have been recently synthesized and characterized by our group, of which we have studied the directionality of intramolecular electron transfer phenomena as a function of the position of the redox centers on the DBF [16].

## 2. Results and Discussion

### 2.1. Synthesis

**Scheme 1:**
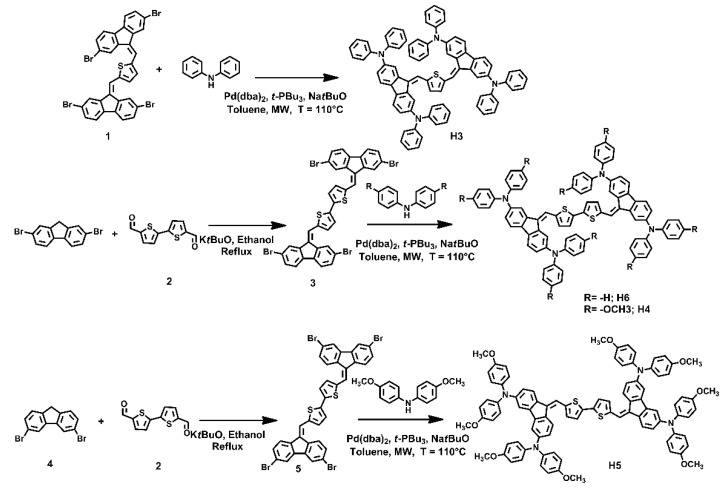
Synthetic route for **H3**, **H4**, **H5** and **H6**.

Synthesis of HTM small molecules was carried out following the straightforward retrosynthetic approach shown in Scheme 1. 

Key steps of the synthetic strategy were the Knoevenagel condensation between fluorene intermediates and thienyl dicarbaldehydes, and the Buchwald–Hartwig cross-coupling reaction between aryl ammines and dibromoaryl derivatives. Both the reactions were carried out in a microwave reactor in very short reaction times and high yields. In detail, bithiophene-2,5-dicarbaldehyde was reacted with 2,7-dibromofluorene or 3,6-dibromofluorene, in the Knoevenagel conditions, using *tert*-BuOK in ethanol at reflux, to yield the corresponding dibromo-intermediates **2** and **4**, respectively. Both compounds were purified by filtration and washing with ethanol. Whereupon, the intermediates **2** and **4** were reacted with diphenylamine or bis(4-methoxyphenyl)amine in the Buchwald–Hartwig conditions, using Pd(dba)_2_ and P(t-Bu)_3_ as the catalyst, *tert*-BuONa as the base, in toluene, to yield the final HTM molecules, **H3**, **H4**, **H5** and **H6**, which were purified by flash chromatography on silica gel.

### 2.2. UV-Vis Properties

**Figure 2:**
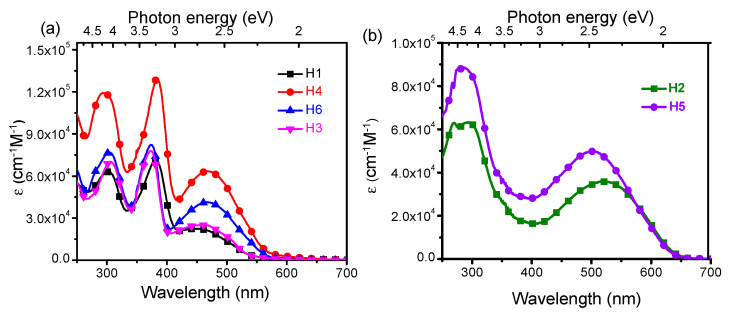
Absorption spectra of (**a**) **H1, H3, H4** and **H6** and (**b**) **H2** and **H5** recorded in CH_2_Cl_2_.

The UV-Vis spectra of **H1–H6** recorded in CH_2_Cl_2_ solution are shown in Figure 2, and the photophysical data are reported in Table 1. The **H1**, **H3,H4** and **H6** spectra show three absorption bands, at 286–307 nm (4–4.3 eV), at 372–384 nm (~3.2–3.3 eV) and at 448–468 nm (~2.6–2.8 eV), while the compounds **H2** and **H5** exhibit only two absorption bands, at 245–307 nm (~4.25–4.3 eV) and at 503–521nm (~2.4–2.5 eV), respectively. In all compounds, the low-energy electron transition can be assigned to the amino-to-bridge charge-transfer character (NB-CT). The results show that the amino substituents contribute differently to the NB-CT depending on their anchoring position on the DBF [17,18]. In fact, in the systems **H1**, **H3**, **H4** and **H6**, the redox centers transfer only partially the charge to the bridge, unlike for the **H2** and **H5** systems where a greater contribution of the amino groups to the charge transfer to the bridge is observed (Figures S2–S4).

The electronic transitions in the range 372–384 nm, for the 2,7-arylamino-fuctionalized, are associated to a π−π* transition of the DBF unit [19], for which the contribution of the redox centers in those specific position is relevant. Indeed, this energy transition is absent in the absorption spectra of **H2** and **H5**. As we reported previously [18], in the latter systems, a larger coupling between the arylamino units with the DBF occurs, which enhances the delocalization of the molecular orbitals on the DBF as well as on the diphenylamine substituents. As a consequence, the DBF π−π* transition is not observed in these systems, but rather a mixed π−π*-CT transition around 265 nm, also involving the diphenylamines. All compounds display an absorption band in the range 295−306 nm assigned to a π−π* transition of the diphenylamine [20]. Peak assignment was performed using single particle transition data obtained from the TD-DFT calculation (see supporting information for more details about the transitions character, Table S1).

### 2.3. Electrochemical and Spectroelectrochemical Characterization

Cyclic voltammograms of the series **H1–H6** compounds exhibit two distinguishable redox processes (Figure S1) between 0 and +1.4 V vs. AgCl/Ag. These correspond to the sequential removal of electrons from the diarylamine moieties and are attributed to the radical cation/neutral (N^▪+/0^) and dication/radical cation (N^++/▪+^) couples (Table 2). All compounds show a large comproportionation constant (Table 2) according to equation KCO=10(ΔE0.059), indicating a good thermodynamic stability of the radical cation species against disproportionation. The coupling among the redox centers as well as the optical energy gap are influenced by the molecular structure differences, essentially determined by the anchoring position of the diarylamine centers on the DBF bridge and, slightly, by the number of thiophenes in the horizontal bridge length. Indeed, even if the potential splitting (ΔE) is large for all the compounds (Table 2), suggesting a strong coupling among the amine centers, that of the 2,7-amine substituted systems is larger than that of the 3,6-amine substituted ones. Moreover, in the systems with one thiophene ring (**H1**, **H2**, **H3**) the splitting is larger, on average, than that measured in the molecules with two thiophene. In particular, inspection of Table 2 shows that in the 2,7-substituted systems, the mono-thiophene bridged ones have systematically larger splitting values than their homologous with two thiophenes (compare **H1** vs. **H4**, and **H3** vs. **H6**); moreover, the splitting difference is highly marked in the 3,6-substituted homologous systems, H2 and H5. The HOMO–LUMO levels were also estimated (Table 2).

**Figure 3:**
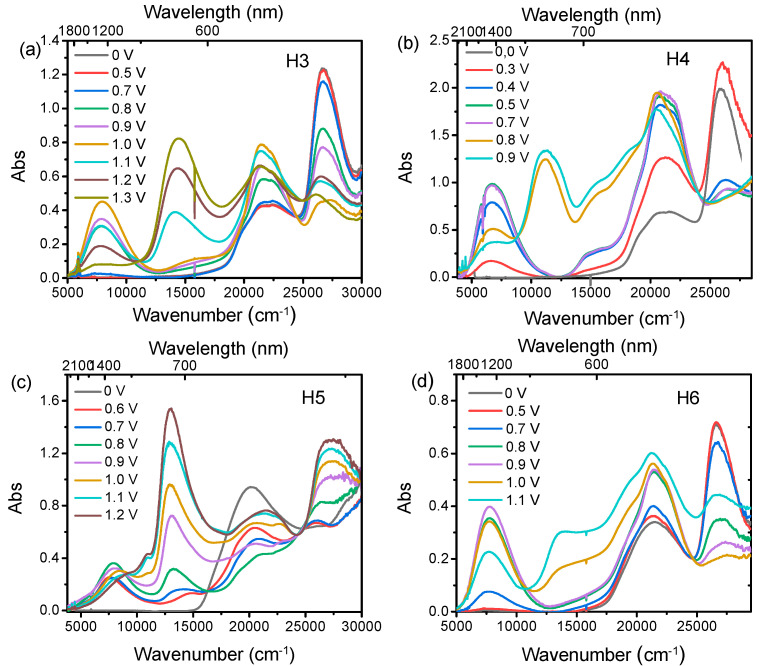
Spectroelectrochemistry of **H3**(**a**), **H4** (**b**), **H5** (**c**) and **H6**(**d**) at c = 10^−3^ M in CH_2_Cl_2_/0.1 M TBAPF_6_, at oxidative potentials.

To gain further insight into the electronic structure of the compounds upon oxidation, spectroelectrochemical experiments in the UV/Vis/NIR region were performed to characterise the mono- and dications (Figure 3). The spectroelectrochemistry of the four compounds (**H3**–**H6**) clearly show that the increase of the oxidative potential causes the growing of a new band in the NIR region (4000–12,000 cm^−1^) and the increase of the absorption at about 22,000 cm^−1^, corresponding to the formation of the arylamino radical cation [21]. The broad band in the NIR region is associated with an optically induced hole transfer, IV-CT, from the oxidized arylamine moiety to the second neutral arylamine moiety belonging to the same DBF unit [16]. Compounds **H3**, **H4**, and **H6,** following the second oxidation, exhibit a similar behaviour, leading to the formation of absorption band centered at 11,088 cm^−1^, 10,914 cm^−1^ and 8856 cm^−1^, respectively, assigned to arylamino-to-bridge charge transition (NB-CT). In particular, in these systems, the IVCT band shape remains unchanged even after the completion of the second oxidation wave, except for a slight moving of the band maximum toward higher energy. On the other hand, the 3,6-substituted **H5** shows different spectroscopic features. When the applied potential is increased positively from 0.6 to 0.8 V (first oxidation process), both the characteristic NIR absorption band at 8000 cm^−1^ and the electron transition NB-CT centered at 13,303 cm^−1^ grow up, due to a significant horizontal electron coupling, as already observed for the 3,6-substituted systems with one thiophene [16].

### 2.4. Interplay between Structure and Optical Properties: Ellipsometry, Raman Spectroscopy and AFM Investigation

**Figure 4:**
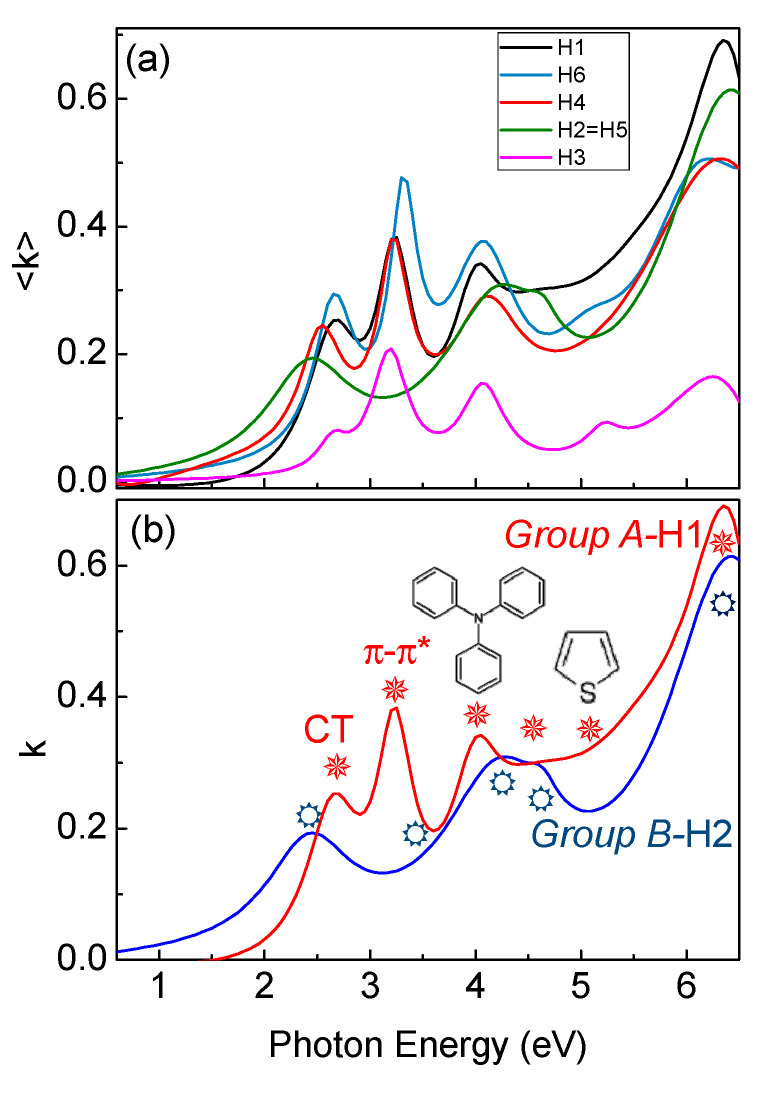
(**a**) Experimental ellipsometric spectra of the pseudoextinction coefficient of all **H1**–**H6**. (**b**) Spectra of the extinction coefficient of Group A and B. Optical transitions are indicated by symbols.

Figure 4a shows the experimental spectra of the pseudoextinction coefficient, <k>, of the various samples. Spectra of **H1**, **H3**, **H4** and **H6** show six main peaks, at 2.6 eV (477 nm) due to the NB-CT transitions, at 3.14 eV (395 nm) related to the π–π* transitions of the conjugated molecule backbones, at 3.99 eV (311 nm),which is characteristic of triphenylamine-derivates [21], at 4.25 eV (292 nm) due to the vinyl-thiophene-derivates, and at 5.24 eV (234 nm) and at 5.91 eV (210 nm), due to thiophene- and benzene-derivates, respectively [22,23]. Spectra of **H2** and **H5**, on the contrary, show that the CT transition is shifted to lower energies, at 2.35 eV (528 nm), while the π–π* transitions of the conjugated molecule backbones is blue-shifted and damped. Moreover, all other transitions in the range 4–6.5 eV (191-310 nm) are also blue-shifted and/or damped.

**Figure 5:**
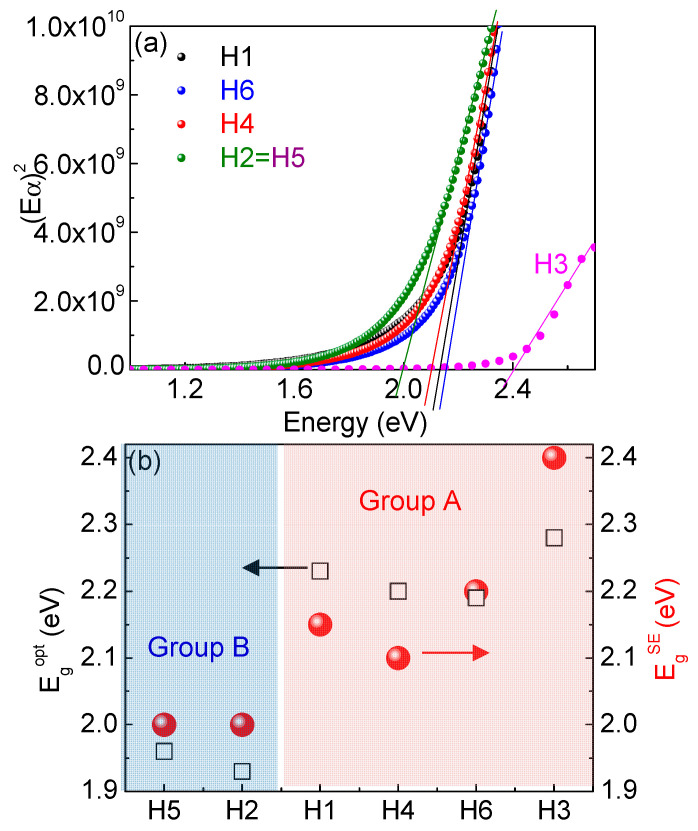
(**a**) (Eα)^2^ vs.energy of different molecules and (**b**) comparison between gaps calculated from ellipsometry ($E_{g}^{opt})$ and by absorption measurements ($E_{g}^{SE})$.

In order to determine energy gaps, we plot (Eα)^2^ as a function of energy, as shown in Figure 5a. We found that **H1**, **H3**, **H4** and **H6** samples have gaps larger than 2 eV, while **H2** and **H5** have gaps lower than 2 eV. Figure 5b shows that there is a good correlation between gaps determined by ellipsometry with gaps in Table 2.

**Figure 6:**
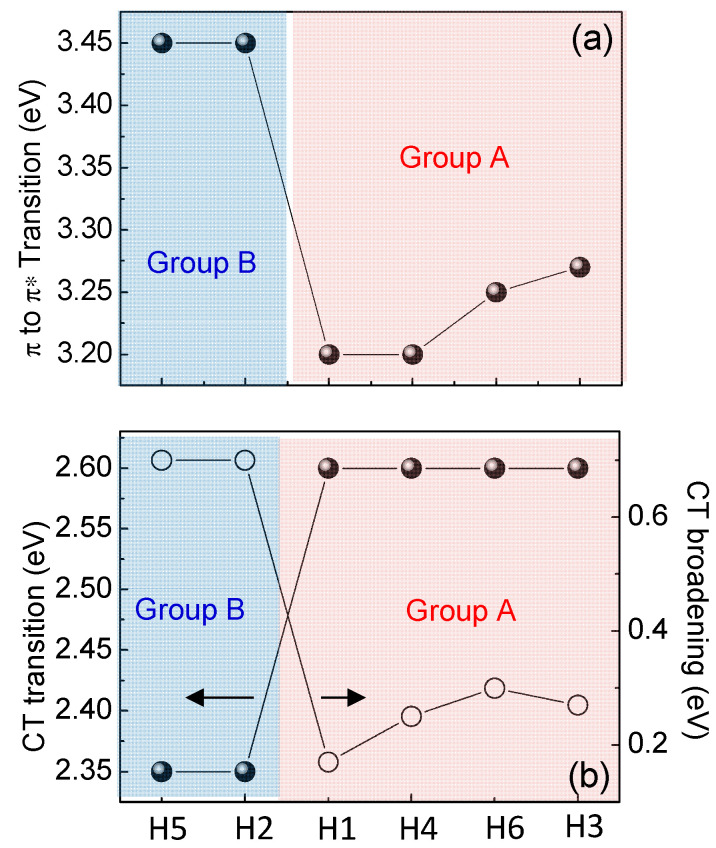
Evolution of the (**a**) π→π* transition and (**b**) position and broadening of the CT transition as a function of molecule type.

All synthesized molecules can be divided in two groups not only considering the different gaps values (Group A includes **H1**, **H3**, **H4** and **H6**, where Group B includes **H2** and **H5**) but also investigating the evolution of the optical transitions in Figure 4b and Figure 6. Figure 4b compares ellipsometric spectra of the extinction coefficient of **H1** as representative of Group A, and **H2** as representative of Group B derived by the ellipsometric fitting, while Figure 6a,b summarizes the evolution of the π–π* transition and of the CT transition in energy and broadening for all the molecules. Specifically, Group A samples (**H1**, **H3**, **H4** and **H6**) have a more red-shifted π–π* transition than Group B molecules (**H2** and **H5**), while Group A CT transition is more-blue shifted with a decrease of its broadening. 

**Figure 7:**
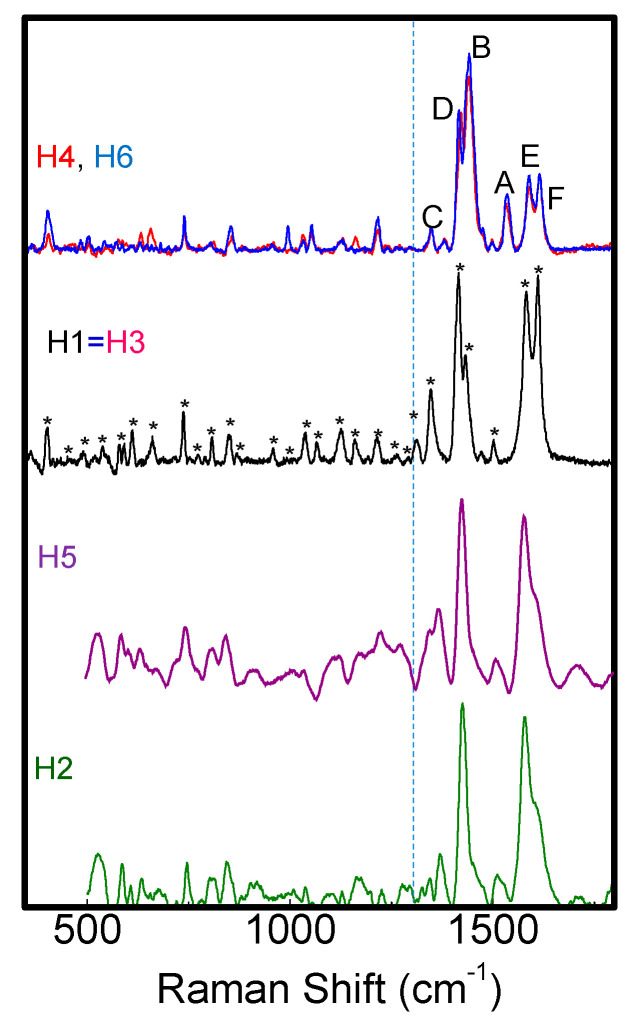
Raman spectra of **H1–H6** samples.

The π–π* red-shift combined with the larger CT intensity and its narrower broadening for Group A samples (**H1**, **H3**, **H4** and **H6**) is indicative of a lower molecule distortion, lower intermolecular interaction (no aggregation) and larger planarity that contribute to a decrease in the structural disorder of the material, compared to Group B compounds (**H2** and **H5**) [24,25,26]. Ellipsometric data have been confirmed by Raman measurements, as shown in Figure 7. 

**Figure 8:**
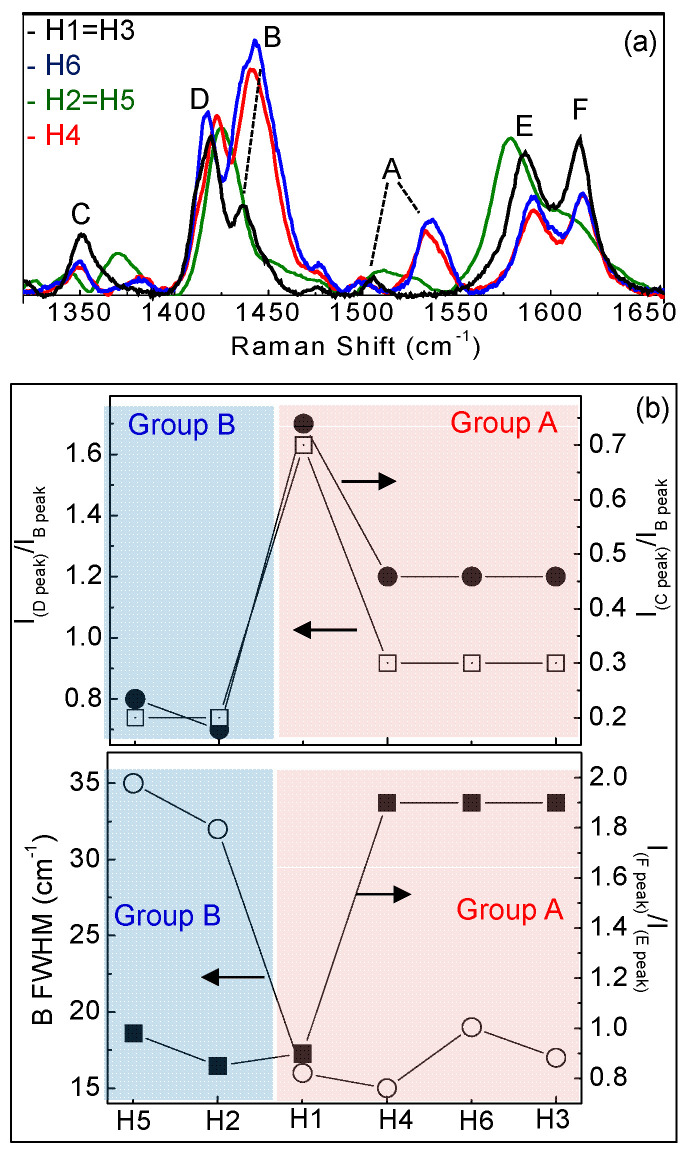
(**a**) Raman spectra in the range 1300–1650 cm^−1^; (**b**) I_Dpeak_/I_Bpeak_ ratio and I_Cpeak_/I_Bpeak_ ratio and I_Fpeak_/I_Epeak_ ratio and full width at half maximum (FWHD) of the B band for **H1**–**H6**.

Raman spectra show typical vibrational modes of C-C, C-C-C, C-H, C-N, C-S-C and C-N-C bonds [27,28,29,30,31,32] correctly assigned in Table S2 and indicated by (*) in Figure 7. At wavenumbers lower than 1300cm^−1^, **H1**–**H6** spectrado not differ significantly; this is different to those atwavenumbers larger than 1300 cm^−1^, as shown in Figure 8a where Raman spectra of **H1**–**H6** in the range 1300–1700 cm^−1^ are compared. 

**Figure 9:**
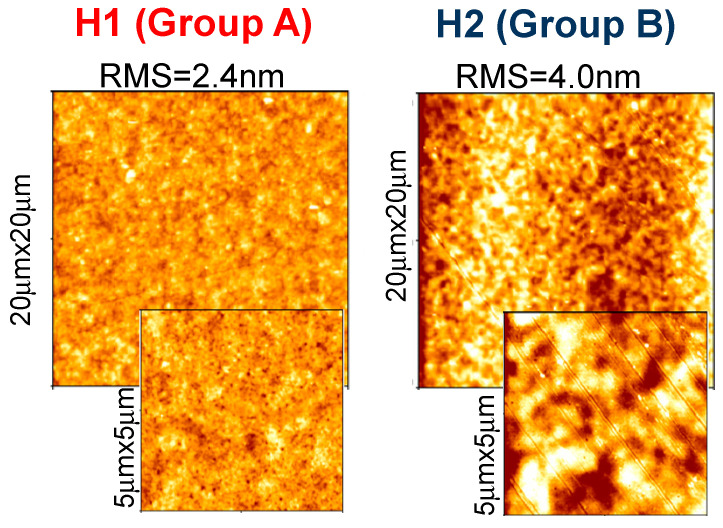
20 μm × 20 μm topographical images, with corresponding zoomed 5 μm × 5 μm ones, of **H1** as representative of Group A, and **H2** as representative of Group B.

We found that the various molecules can be grouped in classes with similar Raman features, consistently with optical results. Group A (that includes molecules **H1**, **H3**, **H4** and **H6**) is characterized by two well resolved intense lines at 1417–1422 and 1437–1443 cm^−1^, labeled as D and B bands in Figure 8a. An additional peak appears in the range 1506–1537 cm^−1^, known as A-line, and assigned to the ring vibration localized at the end rings or to the in-phase antisymmetric ν(C=C) vibration localized on the outer rings of the oligothiophene chain [33]. The region around 1600 cm^−1^ is dominated by two peaks at 1590 and 1615 cm^−1^ of similar intensity, labeled as E and F peaks in Figure 4a, and assigned to C=C vibrations largely localized onto two fluorene moieties, with some contribution from the central thiophene and bithiophene group for the low frequency peak [34]. Conversely, Group B (including **H2** and **H5**) has one single peak at 1431 cm^−1^; the E-F bands are red-shifted to 1579 cm^−1^ and 1608 cm^−1^, with the E-peak being more intense than the F-band. Those differences can be related to molecular order of the solid-state layer, depending on their substituents. Specifically, the thiophene and bithiophene Raman spectra are characterized, respectively, by a single peak at 1408–1425 cm^−1^ [35,36], and at 1416–1443 cm^−1^ [37,38], due to the Cα=Cβ stretching vibration of the thienyl ring, as seen for Group B molecules. This peak splits into two peaks for Group A, one (the D-line) roughly at the same wavenumber of unsubstituted thiophens (in our case ~1420 cm^−1^) that can be assigned to Cα=Cβ-(H) stretching vibration, and the other one (the B-line), at larger wavenumber due to the Cα=Cβ-(substituents) deformation by substitution of the hydrogen terminal of the thiophene ring [39]. This splitting indicates the existence of a vibrational coupling of substituents to the C=C backbone for Group A [31]. Furthermore, a higher intensity of the C-band [40] and D-band [41] with respect to the B-band is indicative of a better molecular order, as seen for the **H1**, which possesses a more planar backbone conformation. In fact, Y. Gao et al. [40] assigned the B-band to unaggregated C=C species in chains with lower intra- and interchain order and with shorter conjugation length. Additionally, a more ordered phase and better crystallinity can also be inferred by the narrower FWHM of the B-band [41,42,43], being 15 cm^−1^ for **H1** and 20 cm^−1^ for **H6** and **H4**. Finally, the relative intensity of the E- and F-band is also useful to evaluate efficient planarity [34]. Specifically, the higher the IF/IE ratio, the higher theplanarity [35], supporting the larger planarity for the **H1** and the lowest planarity for Group B sample **H2** and **H5**. The above considerations about planarity and aggregates of the various molecules also reflect in the different morphology. Figure 9 shows topographical 20 × 20 μm images of **H1** (Group A) and **H2** (Group B) representative samples. Interestingly, the **H1**, as all compounds of Group A, has a smoother morphology with a low surface roughness, whereas the **H2** and **H5** (Group B) show a rougher morphology and less dense structure (the AFM characterization of all compounds are collected in Figure S5 into supporting information).

### 2.5. Space-Charge-Limited Current Hole Mobilities

**Figure 10:**
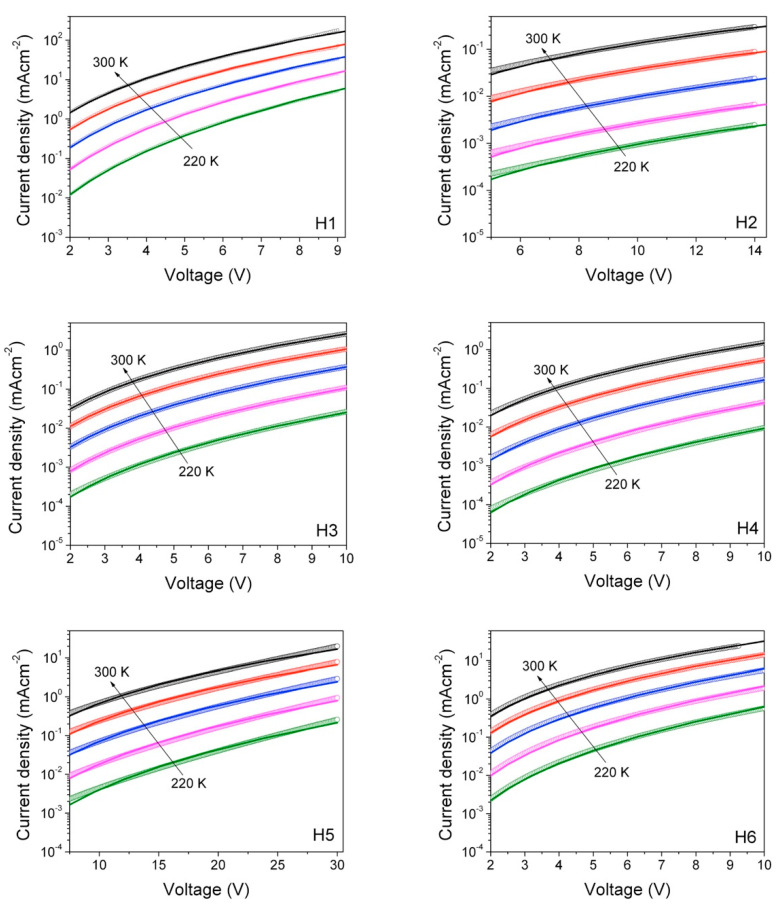
Temperature dependent density current–voltage characteristics of hole-only devices based on the six materials investigated (open circles). The fits to the experimental data (solid lines) are also reported, according to the Murgatroyd-Gill equation.

Unveiling the relationship between materials chemical structure and their charge transport properties is of fundamental importance for their application in optoelectronic devices. For this reason, hole-mobility was measured by the space-charge-limited current (SCLC) method. SCLC is one of the most widely used techniques [44,45] to measure charge mobility in the direction perpendicular to the film, which is the direction most relevant for photovoltaic applications or light emitting diodes [46,47]. Hole-only devices having the following structure: ITO/PEDOT:PSS/Hx/MoO_3_/Au, were realized and characterized under the same experimental conditions, such as in the dark, in a vacuum (10^−3^ mbar) and at a controlled temperature. Figure 10 shows the current density–voltage (J-V) curves in semi-log scale for the six materials investigated (symbols).

**Figure 11:**
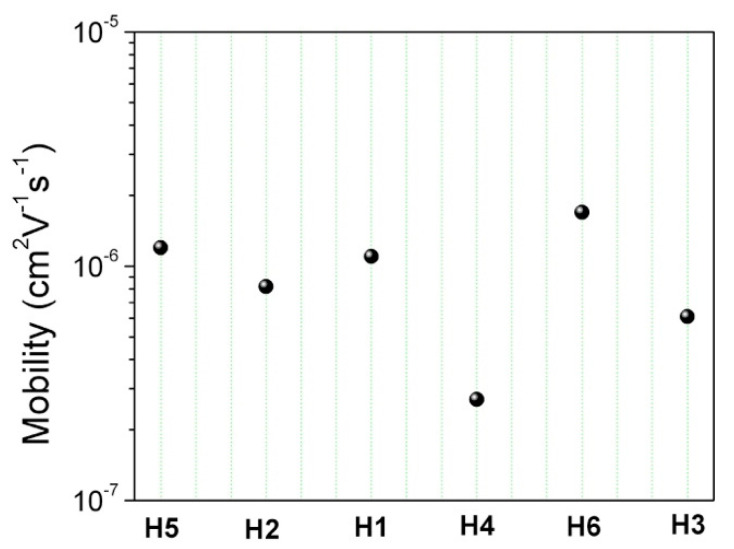
SCLC hole-mobility values for the six materials investigated at zero-field (filled circles).

In order to extract the charge carrier mobility (μ), the J-V curves were analyzed in terms of the space-charge-limited current model, according to the Murgatroyd-Gill Equation (1) (solid line in Figure 10):

(1) JSCL=980ɛɛ0μ0V2L3e0.89VL

where ε_0_ is the permittivity of vacuum, ε_r_ is the relative dielectric constant, assumed to be 3.6, μ_0_ is the zero-field carrier mobility, γ is the field-dependent parameter, and L is the organic film thickness. The estimated zero field mobilities are reported in Figure 11.

To deepen the understanding of charge transport within this series of organic small molecules, we performed SCLC measurements in a temperature range of 220–300 K at a step of 20 K, as seen in Figure 10. Experimental data (symbols) were fitted according to equation (1) along with the Gaussian disorder model (GDM) using the freeware automated analysis tool developed by Kemerink [44], where the only free fitting parameters were μ_0_ and γ, and all the other variables were known. The resulting values of γ were plotted against 1/T (data not shown), and then linearly fitted according to the following Equation (2):

(2) $$\gamma_{\left(T\right)}=B\left[\frac{1}{kT}-\frac{1}{kT_{0}}\right]$$

where B and T_0_ are constant and positive parameters whose resulting values are summarized for each material in Table 3. In a similar way, by plotting μ_0_ against 1/T^2^, we were able to calculate the mobility at zero field and infinite temperature (μ^∗^) and the energetic disorder (σ), by fitting the data according to the following Equation (3):

(3) $$\mu_{0}=\mu^{*}exp\left[-c{\left(\frac{\sigma }{kT}\right)}^{2}\right]$$

Although both groups (A and B) showed only a slight difference in terms of mobility, such feedback on the chemical structure helps to further understand the path to better molecular design.

## 3. Experimental Section

### 3.1. Materials and Methods

All starting materials were purchased from commercial sources and used without further purification. All solvents and reagents were used as received, unless otherwise claimed. 3,6-Dibromo-9*H*-fluorene (**4**), [2,2′-bithiophene]-5,5′-dicarbaldehyde (**2**), 2,5-bis((2,7-dibromo-9*H*-fluoren-9-ylidene)methyl)thiophene (**1**), (*E*)-9-((5-((2-(bis(4-methoxyphenyl)amino)-7-((3-methoxyphenyl)(4-methoxyphenyl)amino)-9*H*-fluoren-9-ylidene)methyl)thiophen-2-yl)methylene)-*N*_2_,*N*_2_,*N*_7_,*N*_7_-tetrakis(4-methoxyphenyl)-9H-fluorene-2,7-diamine (**H1**) and 9,9’-(thiophene-2,5-diylbis(methanylylidene))bis(N_3_,N_3_,N_6_,N_6_-tetrakis(4-methoxyphenyl)-9*H*-fluorene-3,6-diamine) (**H2**) were previously prepared by our group following the synthetic procedure described in the literature [3,7,16]. TLC 0.25 mm silica gel plates with a UV indicator (60F-254, Merck KGaA, Darmstadt, Germany) were used to monitor the progress of reactions. The microwave-assisted syntheses were performed with a CEM Discover Labmate reactor (CEM corporation, CEM SRL, Cologno Al Serio, Italy). ^1^H- and ^13^C NMR spectra were recorded on a Bruker AVANCE III 400 MHz instrument (Bruker Italia SRL, Milano, Italy), using chloroform-*d* (CDCl_3_), acetone-*d*_6_, and dimethylsulfoxide-*d*_6_ (DMSO-*d*_6_) as the solvents, and splitting patterns were described as singlet (s), doublet (d), triplet (t), quartet (q), or multiplet (m). Elemental analyses were done by Carlo Erba CHNS-O EA1108-Elemental Analyzer (CARLO ERBA Reagents Srl, Cornadero (Mi), Italy). LC-MS spectra were acquired with an Agilent 6300 Series Ion Trap source (APCI) (Agilent Technologies Italia Spa, Cernusco sul Naviglio MI, Italy). UV-Vis absorption spectra were recorded on a Varian Cary 3000 spectrophotometer (Agilent Technologies Italia Spa, Italy).

### 3.2. Computational Details

All calculations have been performed using the TURBOMOLE program (TURBOMOLE GmbH, Karlsruhe, Germany) [48,49] using the PBE0-1/3 functional [50] and a def2-TZVP [51] basis set. 

### 3.3. Electrochemistry and Spectroelectrochemistry

Electrochemical characterization of compounds was carried out by cyclic voltammetry (CV) using an AMEL s.r.l. (AMEL s.r.l., Milano, Italy) (Mod. 7050) potentiostat. A typical three-electrode cell was assembled with a glassy carbon disk-working electrode, a Pt-wire auxiliary electrode, and an Ag/AgCl non-aqueous reference electrode [52]. Cyclic voltammograms were acquired at different scan rates, from 50 to 100 mV s^−1^, on 1 mM compound solutions prepared in the electrolyte solution, which consisted of 0.1 M tetrabutylammonium hexafluorophosphate (TBAPF6) in dichloromethane (CH_2_Cl_2_). All the solutions were previously degassed with N_2_. The CV of the ferrocenium/ferrocene (Fc^+^/Fc) couple (0.1 mM) was also recorded in the same condition used for the MV compounds solutions and used as external reference for potential calibration. Spectro-electrochemical experiments were performed with an electrolytic cell (BAS Inc., ALS Co., Ltd, Tokio, Japan) composed of a 1 mm path length cuvette, where a platinum gauze thin layer and a platinum wire were used as the working electrode and the auxiliary electrode, respectively. A pseudo-reference electrode consisting of an Ag wire was calibrated against the Fc+/Fc redox couple [53]. The spectro-electrochemical cell was filled with dichloromethane solutions of each compound (from 1 to 0.25 mM) and TBAPF6 (0.1 M). UV-vis-NIR spectra were recorded using a Vertex 80 (Bruker Italia SRL, Milano, Italy)) spectrophotometer. The potential was supplied by means of an Amel 2049 model potentiostat. Measurements were performed at 25 °C.

### 3.4. Spectroscopic Ellipsometry, Ramanand AFM Characterization

Spectroscopic ellipsometry spectra of the refractive index, n, and extinction coefficient, k, were measured in the 1.5–6.5 eV energy range by use of a phase modulated spectroscopic ellipsometer (UVISEL, Jobin Yvon, HORIBA Ltd, Palaiseau, France) at an incidence angle of 70.00.Parameterization of optical constants in the UV-Vis range is based on the combination of Lorentzian oscillators to take into account the contribution of band-to-band transitions. The number of oscillators depends on the band structure of the semiconductor. For the present molecules, six Lorentz oscillators were used. Experimental SE spectra were fitted using a two-layer model, air/molecule/substrate, where fit variables were the dispersion equation and film thickness. The total film thickness was also measured by a profilometer (alpha step). An isotropic model was assumed in the data analysis, since previous measurements on films from spin-coating with the thickness in the range investigated in the present paper did not show significant anisotropy. Films were measured with a 1.5 mm^2^ spot in different points of the samples and all SE measurements for a sample were fit to the same model and dispersion equation; differences were within the 95% confidence limit of fit parameters, indicating a good lateral homogeneity of samples. 

Raman spectra were collected using a LabRAM HR Horiba-Jobin Yvon spectrometer with the 532 nm excitation lasers. The combination of Ellipsometric and Raman analysis was useful to correlate optical properties (spectra of extinction coefficient, energy gap and energy position of optical transitions, intensity and width of Raman bands) with structural/conformational properties, like planarity, distortion and aggregation, as a function of the molecule structure and substituents. 

Atomic force microscopy (AFM), measurements were performed in non-contact mode to characterize the surface morphology by Autoprobe CP (ThermoMicroscopes, Sunnyvale, CA, USA).

### 3.5. Fabrication and Characterization of Hole-Only Devices

Hole-only devices were fabricated by spin-coating in air onto precleaned and O_2_-plasma-treated ITO patterned glass substrates, a 40 nm thin film of polystyrene sulfonate (PEDOT:PSS) at 4000 rpm for 60 s, followed by 20 min thermal annealing at 140 °C. The HTM was spin-coated in a glovebox from a chloroform solution at a concentration of 70 mg/mL and at 2000 rpm, which led to a film thickness in the range of 500 nm. A 10 nm MoO_3_, followed by 80 nm Au, were deposited as the top electrode by high-vacuum (10^−6^ mbar) thermal evaporation. The device areas were measured by an optical microscope, and they were in the range of 0.035−0.045 cm^2^. SCLC measurements were performed at room temperature in the dark, loading the sample into a cryostat under low dynamic vacuum (10^−3^ mbar). Current−voltage characteristics were recorded using a computer-controlled pico-ammeter (HP 4140B), by applying a positive bias to the bottom electrode (ITO/PEDOT). Data analysis for charge transport was performed using an automated open-source software developed by Kemerink [44].

## 4. Conclusions

Two classes of materials, for a total of six different molecules, were synthesized with an H-shape design, based on a molecular structure consisting of two D-A-D units connected by a thiophene or bitiophene bridge, and the diarylamino donor units anchored to the 2,7- and 3,6 positions of the dibenzofulvene backbone. Optical, electrochemical, morphological and hole-transporting properties were investigated, by UV-Vis spectroscopy, ellipsometry, Raman spectroscopy, AFM microscopy, cyclic voltammetry, TD-DFT calculations and SCLC method. In this way, we have elucidated the differences between the two classes of molecules and investigated the role of the molecular design on the various properties. All the compounds exhibited reversible redox properties and possess a low oxidation potential aiding facile removal of an electron from the HOMO, as well as the propensity to form a stable radical form. Furthermore, they showed good solubility, and chemical and environmental stability. In addition, Group A materials showed a lower structural disorder, larger planarity, and better flatness compared to Group B ones.

Although the SCLC measurements did not show high hole-mobility values for any of the studied compounds, and it was not possible to record a significant correlation between the hole-mobility measurements and the chemical structure, Group A could be identified as the one with the most promising characteristics for the development of hole-transporting materials based on the DBF structure, according to structural and morphological characterization. This study demonstrated therefore a rational tailoring of the diphenylamine-based electron donors on the dibenzofulvene backbone, in order to fine tune the optoelectronic, structural and morphological properties. The versatility of the DBF reactive sites will allow us to introduce a wide variety of arylamino groups to improve their morphological and electric properties, in order to obtain promising hole transport materials.

## Figures and Tables

**Table 1:** Experimental absorption energies and molar absorption coefficients, as well as absorption energies, oscillator strengths (o.s) and transitions character from TD-DFT simulations.

Sample	λ_exp-__abs_/nm	ε/M^−1^cm^−1^	λ_TD-DFT-__abs_/nm	o.s.	Character
**H1**	448	22,465	434	0.31	NBCT
	381	71,308	376	0.85	π-π* DBF
	303	63,061	289	0.78	π-π* Arylamine
**H2**	521	35,811	489	0.46	NBCT
	291	63,190	288	0.66	π-π* DBF
**H3**	458	25,127	441	0.30	NBCT
	372	77,760	388	0.87	π-π* Arylamine
	305	70,093	282	0.73	CT
**H4**	468	63,453	428	0.37	NBCT
	384	129,198	385	0.66	π-π* Arylamine
	295	119,205	2.90	0.89	CT
**H5**	503	49,865	472	0.51	NBCT
	286	88,504	278	0.72	π-π* Arylamine
**H6**	466	41,384	444	0.45	NBCT
	375	82,131	356	0.79	π-π* DBF
	305	76,727	282	0.68	π-π* Arylamine

**Table 2:** Electrochemical properties and corresponding energy levels ^a^.

Sample	E_1/2_(1) (mV))	E_1/2_(2) (mV)	^b^ ΔE_1_ (mV)	^c^ E_HOMO_ (eV)	^d^ E_LUMO_ (eV)	^e^$E_{g}^{opt}$ (eV)
**H1**	72	347	275	−5.20	−2.97	2.23
**H2**	47	374	326	−5.23	−3.3	1.93
**H3**	248.8	536.2	287.4	−5.28	−2.98	2.28
**H4**	45.8	308.8	263	−5.11	−2.9	2.20
**H5**	138.2	334	196.6	−5.22	−3.26	1.96
**H6**	245.6	505.2	259.6	−5.3	−3.1	2.19

^a^ c = 10^−3^ mol/L in CH_2_Cl_2_/TBAPF_6_ (0.1 M) vs. Fc^+^/Fc at 50 mV/s. ^b^ ΔE = E_1/2_(2) − E_1/2_(1). ^c^ E_HOMO_ = −(E_onset_(ox1) + 5.16) (eV). ^d^ E_LUMO_ = *E_g_^opt^* − E_HOMO._^e^ The bandgap $E_{g}^{opt}$ was determined from onset of the absorption spectrum.

**Table 3:** Fitting parameters for hole-only devices of Figure 9, extracted from the MG+GDM model.

Sample	μ_0_ @ 300K (cm^2^/Vs)	σ (meV)	B	μ^∗^ (cm^2^Vs)	T0 (K)	γ (V/m)
**H6**	1.7 × 10^−6^	102	2.6 × 10^−5^	1.5 × 10^−3^	480	3.7 × 10^−4^
**H4**	2.7 × 10^−7^	105	2.0 × 10^−5^	3.1 × 10^−4^	983	5.2 × 10^−4^
**H3**	6.1 × 10^−7^	104	2.4 × 10^−5^	8.2 × 10^−4^	1031	6.1 × 10^−4^
**H2**	8.2 × 10^−7^	113	1.3 × 10^−5^	4.0 × 10^−4^	492	2 × 10^−4^
**H5**	1.2 × 10^−6^	104	2.2 × 10^−5^	1.4 × 10^−3^	557	4 × 10^−4^
**H1**	1.1 × 10^−6^	98	2.7 × 10^−5^	5.2 × 10^−4^	553	4.9 × 10^−4^

## Data Availability

Not applicable.

## Supplementary Materials

The following are available online: the synthetic procedures, cyclic voltammetry, isodensity plots of the frontier molecular orbitals of **H1**–**H6**, and ^1^H-NMR and ^13^C-NMR spectra.

## Sample Availability

Samples of the compounds are not available from the authors.
