# Supplementary material for: Synthesis and Investigation of Electro-Optical Properties of H-Shape Dibenzofulvene Derivatives
Source: Molecules. 2022 Feb 6;27(3):1091. doi: 10.3390/molecules27031091 (PMC8838082; doi:10.3390/molecules27031091)
Supplement: Supplementary file 1 [file molecules-27-01091-s001.zip › molecules-1553705-supplementary.pdf]

Supporting Information

Synthesis and Investigation of Electro-Optical Properties of H-Shape Dibenzofulvene Derivatives

Maria Michela Giangregorio [1], Salvatore Gambino [2], Eduardo Fabiano [3,4], Mauro Leoncini [2,5], Antonio Cardone [6], Giuseppina Anna Corrente [7], Amerigo Beneduci [7], Gianluca Accorsi [2], Giuseppe Gigli [2,5], Maria Losurdo [1], Roberto Termine [8] and Agostina-Lina Capodilupo [2,*]

[1] Institute of Nanotechnology (CNR-NANOTEC), c/o Department of Chemistry, University of Bari, Via Orabona 4, 70126 Bari, Italy; michelaria.giangregorio@nanotec.cnr.it (M.M.G.); maria.losurdo@cnr.it (M.L.)
[2] Institute of Nanotechnology (CNR-NANOTEC), c/o Campus Ecotekne , University of Salento, via Monteroni, Lecce 73100, Italy; salvatore.gambino@nanotec.cnr.it (S.G.); mauro.leoncini@nanotec.cnr.it (M.L.); gianluca.accorsi@nanotec.cnr.it (G.A.); giuseppe.gigli@nanotec.cnr.it (G.G.)
[3] Institute for Microelectronics and Microsystems (CNR-IMM), c/o Campus Ecotekne , University of Salento, via Monteroni, Lecce 73100, Italy; eduardo.fabiano@cnr.it
[4] Centre for Biomolecular Nanotechnologies @UNILE, Istituto Italiano di Tecnologia (IIT), Lecce 73010, Italy
[5] Dipartment of Mathematics and Physics "Ennio de Giorgi", University of Salento, Lecce 73100, Italy
[6] Institute of Chemistry of OrganoMetallic Compounds, ICCOM, Italian National Council of Research, CNR, Via Orabona 4, 70125, Bary, Italy; cardone@ba.iccom.cnr.it
[7] Department of Chemistry and Chemical Technologies, University of Calabria, Via P. Bucci, Cubo 15D, 87036 Arcavacata di Rende (CS), Italy; amerigo.beneduci@unical.it (G.A.C.); giuseppina.corrente@unical.it (A.B.)
[8] Institute of Nanotechnology (CNR-NANOTEC), c/o University of Calabria, Via P. Bucci, 87036 Rende (CS), Italy; roberto.termine@cnr.it
* Correspondence: agostina.capodilupo@nanotec.cnr.it

Contents

**Synthetic Procedures**	**S3**
**Cyclic Voltammetry**	**S6**
**Isodensity plots of the frontier molecular orbitals of H1-H6**	**S7**
**Assignment of Raman bands**	**S11**
**Topography by Atomic force microscopy**	**S13**
**Nuclear magnetic resonance spectra**	**S14**

Synthetic Procedures

9,9'-(thiophene-2,5-diylbis(methanylylidene))bis(N2,N2,N7,N7-tetraphenyl-9H-fluorene-2,7-diamine), H3.

A mixture of 2,5-bis((2,7-dibromo-9H-fluoren-9-ylidene)methyl)thiophene (**1**) (0.400 g, 0.532 mmol), diphenylamine (0.389 g, 2.3 mmol) and sodium *tert*-butoxide (0.265 g, 2.76 mmol) was added to a suspension of Pd(dba)2 (0.025 g, 0.043 mmol) and P*t*Bu3 (0.174 mL, 0.174 mmol, 1M in toluene) in anhydrous and deoxygenated toluene (10 mL), previous stirred under argon for 10 min. The resulting solution was heated under microwave irradiation at a constant temperature of 110°C for 60 min. The solvent was removed, and the residue was extracted in dichloromethane. The organic phase was washed with brine, dried over anhydrous Na_2SO_4 and concentrated by rotary evaporation. The crude residue was purified by column chromatography on silica gel, using a mixture of hexane/CH2Cl2 (7/3 v/v) as the eluent, to give the pure product as a red solid (82% yield). ^{1}H NMR (400 MHz, $CDCl_3$) δ 7.89 (s, 2H), 7.52-7.48 (m, 6H), 7.30-7.26 (m, 6H), 7.17-7,00 (m, 36H), 6.87-6.83 (m, 4H), 6.75 (s,2H).^{13}C NMR (101 MHz, $CDCl_3$) δ 147.79, 147.55, 146.59, 146.32, 141.02, 140.44, 136.97, 135.34, 135.09, 134.12, 130.38, 129.11, 129.05, 125.45, 124.28, 124.10, 124.01, 123.78, 123.54, 122.64, 122.51, 122.32, 120.66, 119.73, 119.59, 118.99, 116.70. MS (APCI): m/z 1105,39 calcd. for $C_{80}H_{56}N_4S$: found: m/z = 1106,40 $[M+H]^+$. Elemental Analysis calcd for $C_{80}H_{56}N_4S$: C, 86.92; H, 5.11; N, 5.07; S, 2.90; found: C, 86.83; H, 5.22; N, 5.03; S, 2.88.

Synthesis of 5,5'-bis((2,7-dibromo-9H-fluoren-9-ylidene)methyl)-2,2'-bithiophene, 3:

2,7-dibromo-9Hfluorene (1.00 g, 3.1 mmol), [2,2'-bithiophene]-5,5'-dicarbaldehyde (0.344 g, 1.55 mmol) and potassium *tert*-butoxyde (0.520 g, 4.65 mmol) were dissolved in 15 mL of absolute ethanol, and reacted in an ultrasound bath for 15 minutes. An orange precipitate was obtained, which was filtered and washed several times with ethanol (90% yield). The resulting product has a very low solubility in the common organic solvents, and was used, without further purification, in the next reaction.

9,9'-([2,2'-bithiophene]-5,5'-diylbis(methanylylidene))bis(N2,N2,N7,N7-tetrakis(4-methoxyphenyl)-9H-fluorene-2,7-diamine), H4

A mixture of **3** (0,400 g, 0.482 mmol), bis(4-methoxyphenyl)amine (0.486 g, 2.12 mmol) and sodium *tert*-butoxide (0.214 g, 2.23 mmol) was added to a suspension of $Pd(dba)_2$ (0.022 g, 0,038 mmol) and P*t*Bu3 (0.152 mL, 0.152 mmol, 1M in toluene) in anhydrous and deoxygenated toluene (10 mL),

previous stirred under argon for 10 min. The resulting mixture was heated under microwave irradiation at a constant temperature of 110°C for 60 min. The solvent was removed under vacuum and the residue was extracted with dichloromethane. The organic phase was washed with brine, dried over anhydrous Na_2SO_4 and concentrated by rotary evaporation. The residue was purified by column chromatography on silica gel, using a mixture of hexane/CH_2Cl_2 (5/5 v/v) as the eluent, to give the pure product as a red solid (77% yield). ^{1}H NMR (400 MHz, DMSO-d_6) δ 7.85 (d, J = 2.1 Hz, 2H), 7.55 (d, J = 9.1 Hz, 2H), 7.52-7.50 (m, 3H), 7.44 (d, J = 1.9 Hz, 2H), 7.27 (d, J = 4.4 Hz, 2H), 7.04-6.95 (m, 16H), 6.90-6.84 (m, 20H), 6.81 (d, J = 8.2 Hz, 4H), 3.72 (s, 12H), 3.61 (s, 12H). ^{13}C NMR (101 MHz, $CDCl_3$)157.47, 156.75, 146.49, 146.30, 140.91, 138.47, 137.95, 137.05, 135.60, 135.04, 129.44, 125.44, 124.41, 123.94, 123.54, 122.48, 122.25, 120.79, 119.72, 118.81, 117.61, 116.73, 55.81. MS (APCI): m/z 1427,72 calcd. for $C_{92}H_{74}N_4O_8S_2$: found: m/z = 1428,82 $[M+H]^+$. Elemental Analysis calcd for $C_{92}H_{74}N_4O_8S_2$ C, 77.40; H, 5.22; N, 3.92; O, 8.96; S, 4.49; found: C, 77.62; H, 5.26; N, 3.97; S, 4.41.

9,9'-([2,2'-bithiophene]-5,5'-diylbis(methanylylidene))bis(N2,N2,N7,N7-tetraphenyl-9H-fluorene-2,7-diamine), H6

Following the same procedure of **H4,** a mixture of **3** (0,400 g, 0.482 mmol), diphenylamine (0.358 g, 2.12 mmol) and sodium *tert*-butoxide (0.214 g, 2.23 mmol) was added to a suspension of $Pd(dba)_2$ (0.022 g, 0,038 mmol) and P*t*Bu_3 (0.152 mL, 0.152 mmol, 1M in toluene) in anhydrous and deoxygenated toluene (10 mL), previous stirred under argon for 10 min. The resulting mixture was heated under microwave irradiation at a constant temperature of 110°C for 60 min. The solvent was removed, and the residue was extracted with dichloromethane. The organic phase was washed with brine, dried over anhydrous Na_2SO_4 and concentrated by rotary evaporation. The residue was purified by column chromatography on silica gel. Using a mixture of hexane/CH_2Cl_2 (6/4 v/v) as the eluent, to give the pure product as a red solid (85% yield). ^{1}H NMR (400 MHz, $CDCl_3$) δ 7.98 (s, 2H), 7.53-7.50 (m, 4H), 7.30-7.27 (d, J = 8.3 Hz, 8H), 7.26-7,20 (m, 11H), 7.15-7.09 (m, 18H), 7.04-6.94 (m, 11H), 6.64 (d, J = 3.8 Hz, 2H). ^{13}C NMR (101 MHz, $CDCl_3$) δ 147.75, 147.62, 146.49, 146.30, 140.91, 138.47, 137.95, 137.05, 135.60, 135.04, 134.04, 130.74, 129.05, 125.44, 124.41, 123.94, 123.54, 122.48, 122.25, 120.79, 119.72, 118.81, 117.61, 116.73. MS (APCI): m/z 1187,52calcd. for $C_{84}H_{58}N_4S_2$: found: m/z = 1188,50 $[M+H]^+$. Elemental Analysis calcd for $C_{84}H_{58}N_4S_2$: C, 84.96; H, 4.92; N, 4.72; S, 5.40; found: C, 85.02; H, 5.04; N, 4.59; S, 5.67.

5,5'-bis((3,6-dibromo-9H-fluoren-9-ylidene)methyl)-2,2'-bithiophene, 5

3,6-dibromo-9Hfluorene (1.00 g, 3.1 mmol), [2,2'-bithiophene]-5,5'-dicarbaldehyde (0.344 g, 1.55 mmol) and potassium *tert*-butoxyde (0.520 g, 4.65 mmol) were dissolved in 15 mL of of absolute ethanol. The mixture was reacted in an ultrasound bath for 15 minutes, and the resulting orange precipitate was filtered and washed several times with ethanol (92% yield). The product has a very low solubility in the common organic solvents, and was used, without further purification, in the next reaction.

9,9'-([2,2'-bithiophene]-5,5'-diylbis(methanylylidene))bis(N3,N3,N6,N6-tetrakis(4-methoxyphenyl)-9H-fluorene-3,6-diamine), H5

A mixture of **5** (0,400 g, 0.482 mmol), bis(4-methoxyphenyl)amine (0.486 g, 2.12 mmol) and sodium *tert*-butoxide (0.214 g, 2.23 mmol) was added to a suspension of $Pd(dba)_2$ (0.022 g, 0,038 mmol) and P*t*Bu_3 (0.152 mL, 0.152 mmol, 1M in toluene) in anhydrous and deoxygenated toluene (10 mL), previous stirred under argon for 10 min. The resulting mixture was heated under microwave irradiation at a constant temperature of 110°C for 60 min. The solvent was removed under vacuum, and the residue was extracted with dichloromethane. The organic phase was washed with brine, dried over anhydrous Na_2SO_4 and concentrated by rotary evaporation. The residue was purified by column chromatography on silica gel, using a mixture of hexane/CH_2Cl_2 (2/8 v/v) as the eluent, to give the pure product as a purple solid (80% yield). ^{1}H NMR (400 MHz, DMSO-d_6) δ 8.13 (d, J = 8.6 Hz, 2H), 7.69 (d, J = 8.5 Hz, 2H), 7.47 (s, 2H), 7.42-7.40 (m, 3H), 7.03-6.97 (m, 16H), 6.90-6.86 (m, 21H), 6.70 (dd, J = 8.4, 2.1 Hz, 2H), 6.62 (dd, J = 8.7, 2.2 Hz, 2H), 3,72 (s, 24H). ^{13}C NMR (101 MHz, DMSO) δ 156.26, 155.89, 149.39, 149.05, 141.79, 140.55, 140.02, 139.11, 138.84, 137.38, 133.97, 133.44, 132.17, 128.10, 127.10, 126.52, 125.31, 121.80, 119.95, 118.26, 115.21, 114.83, 111.08, 110.27, 55.47. MS (APCI): m/z 1427,72 calcd. for $C_{92}H_{74}N_4O_8S_2$: found: m/z = 1428,84 $[M+H]^+$. Elemental Analysis calcd for $C_{92}H_{74}N_4O_8S_2$: C, 77.40; H, 5.22; N, 3.92; O, 8.96; S, 4.49; found: C, 77.61; H, 5.22; N, 3.80; S, 4.37.

Electrochemistry

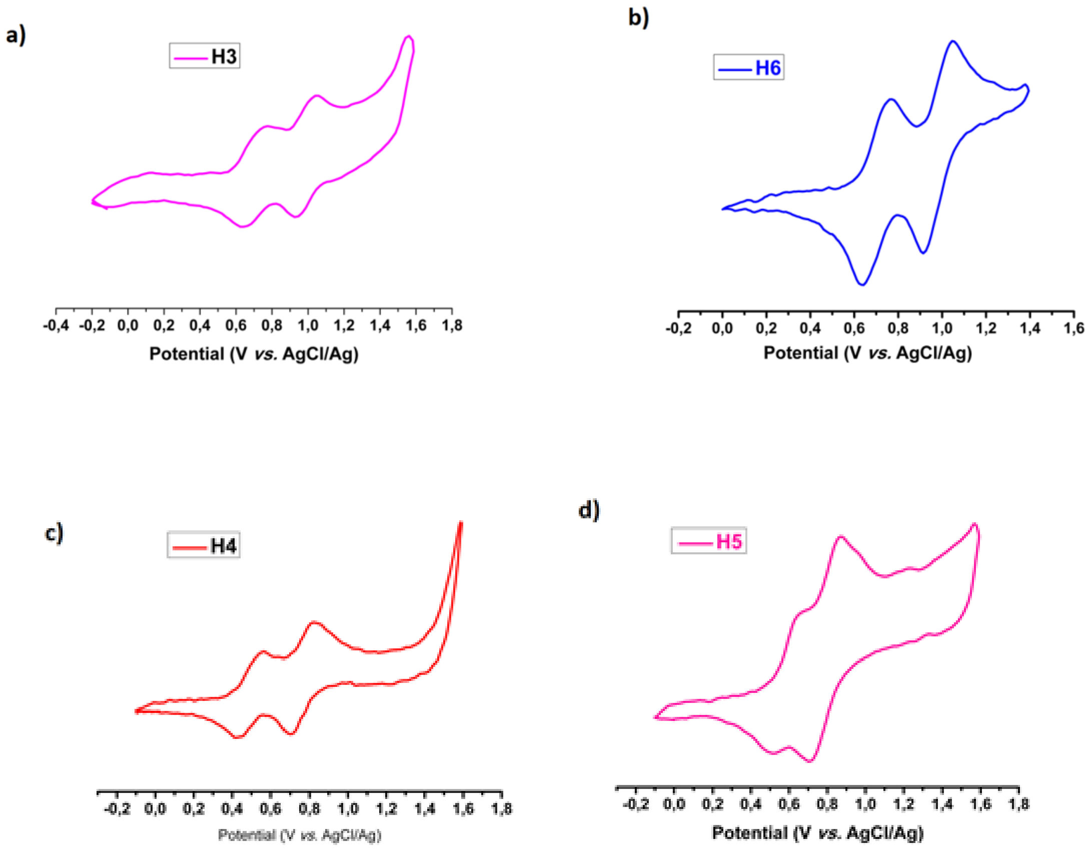

Figure S1: Cyclic voltammetry of compounds **H3**, **H4**, **H5** and **H6**, at c = 10^{-3} M in $CH_2Cl_2/TBAPF_6$ (0.1 M) *vs* Ag/AgCl.

Isodensity plots of the frontier molecular orbitals of H1-H6.

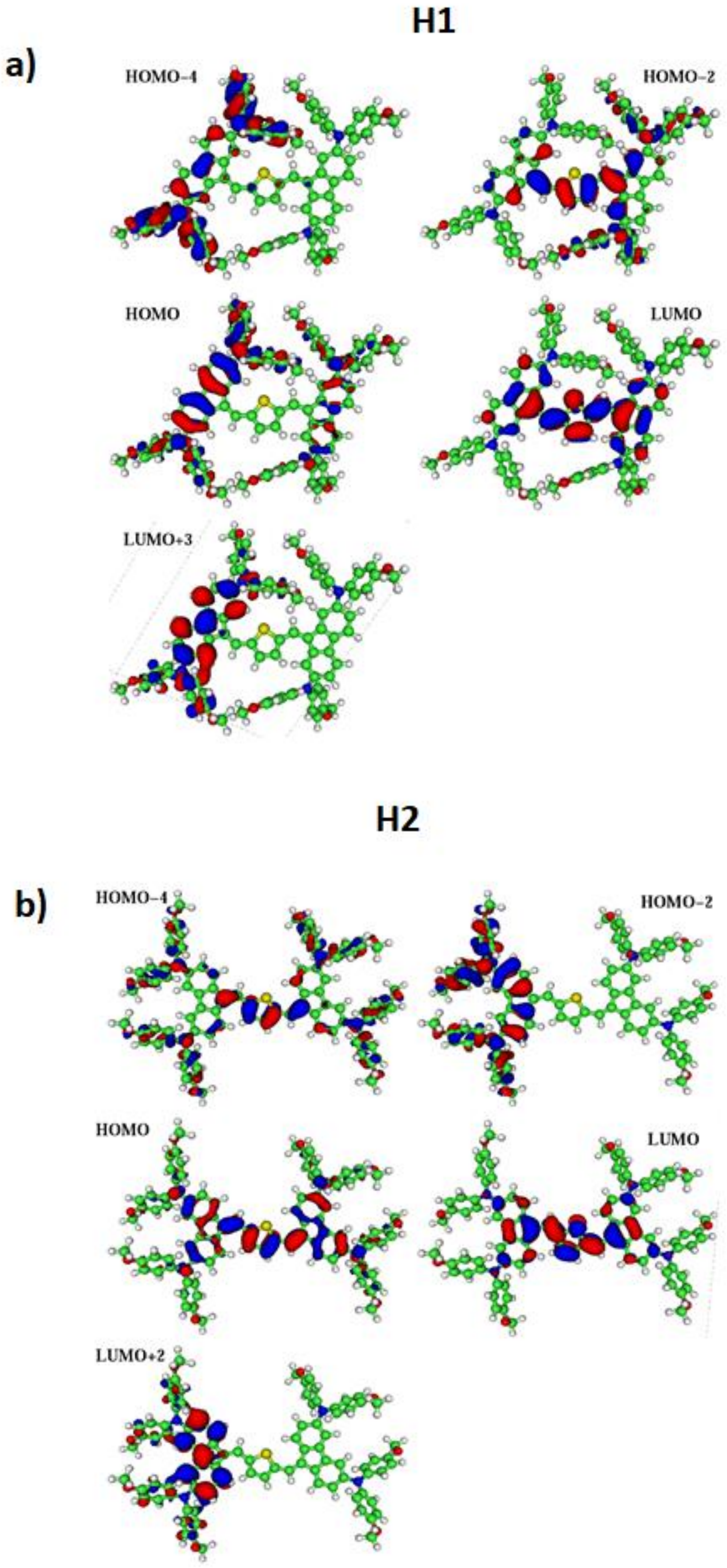

Figure S2: Isodensity plots of the frontier molecular orbitals of **H1** and **H2**.

H3

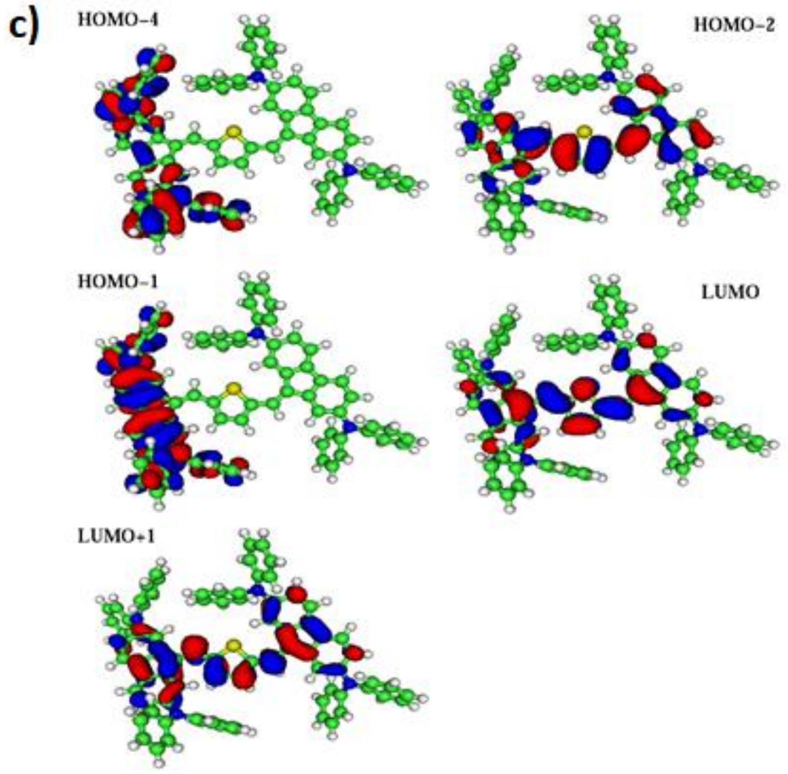

H4

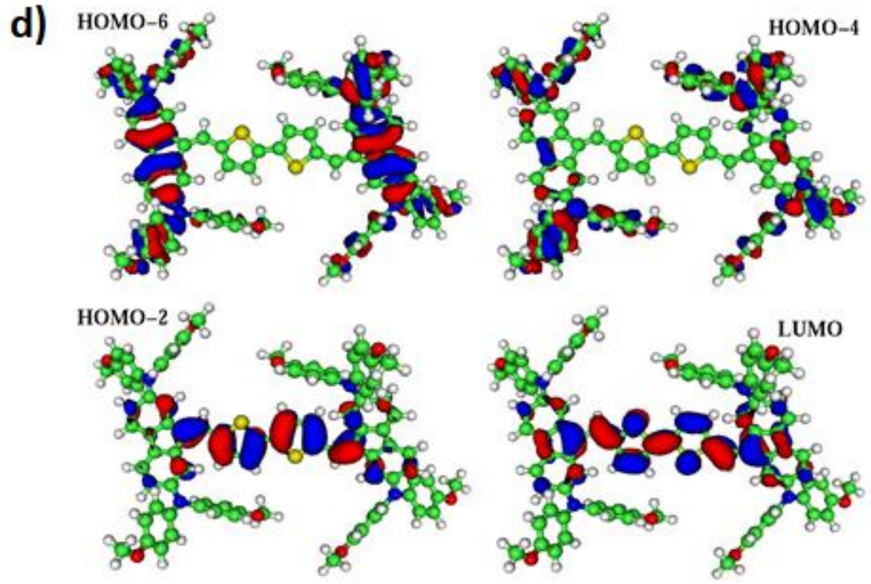

Figure S3: Isodensity plots of the frontier molecular orbitals of **H3** and **H4.**

H5

e)

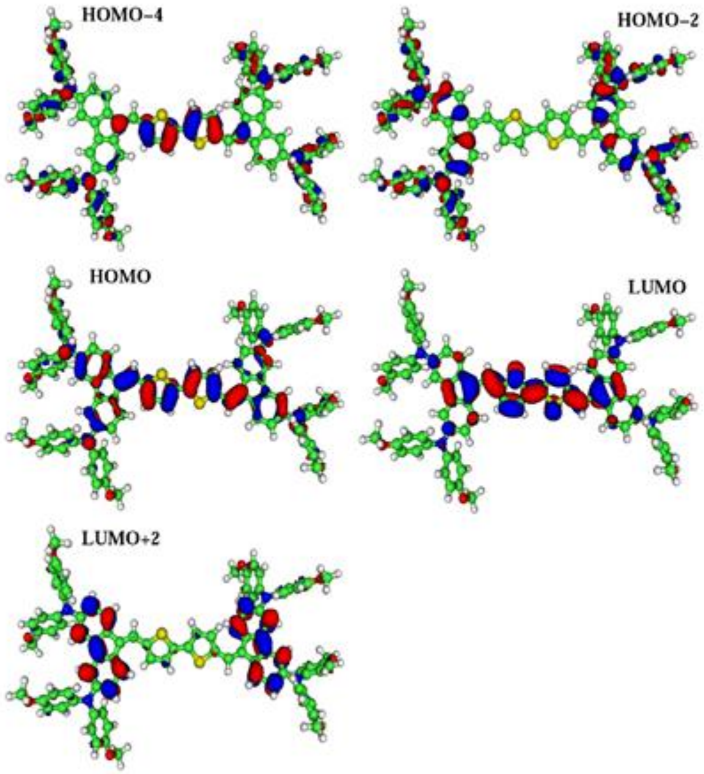

H6

f)

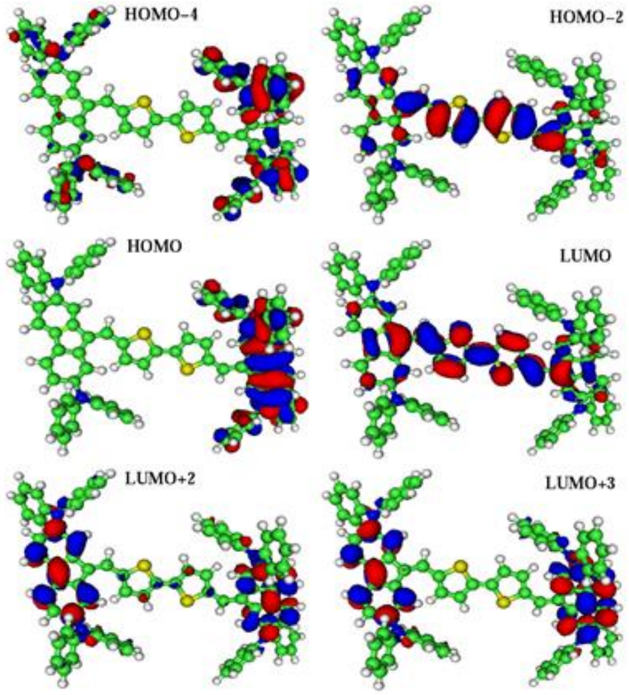

Figure S4: Isodensity plots of the frontier molecular orbitals of **H5** and **H6.**

Table S1: Main single particle transitions for the most relevant electronic excitations of Table 1.

**Sample**	**Sing. Part. Transition**
**H1**	H-4 → L
	H → L+3
**H2**	H-2 → L
	H → L
**H3**	H-4 → L
	H-4 → L +2
**H4**	H-6 → L
	H-4 → L +1
**H5**	H-2 → L
	H-2 → L +1
**H6**	H-4 → L
	H → L +2

Assignment of Raman bands

Table S2: Assignment of Raman peaks shown in Figure 7.

Raman peak (cm^{-1})	Peak assignment
**388**	Ring torsion
**417**	Ring torsion & deformation
**485**	Ring torsion & deformation<br>CNC deformation
**558**	Ip ring bending
**584**	Ip ring bending
**635**	ring torsion<br>ip (CH+CCC) bending
**715**	oop CH and CCC bending<br>CH2 rocking
**729**	oop CH bending
**740**	C-S-C deformation
**780**	Oop and ip CH and CCC bending<br>CNC/CSC deformation<br>CH ring wagging
**809**	oop CH and CCC bending<br>CH ring wagging
**833**	Oop N-H wagging<br>CS ring stretching
**910**	oop CH and CCC bending<br>CH2 rocking<br>CH ring wagging
**956**	Ring breathing<br>ip (CH+CCC) bending<br>CH ring wagging
**1073**	Ip(CH+CCC) bending<br>CC stretching
**1124**	Ip (CH+CCC) bending<br>CNC stretching
**1167**	Ip (CH+CCC) bending<br>CN stretching
**1190**	Ip (CH+CCC) bending<br>CH deformation
**1217**	Ip (CH+CCC) bending<br>Ring stretching
**1243**	ring stretching<br>ip CH bending<br>CN stretching
**1268**	Ip (CH+CCC) bending<br>CH2 wagging
**1295**	Ip (CH+CCC) bending<br>CC stretching

**1305**	Ip (CH+CCC) bending<br>CH2 wagging<br>CC stretching
**1346 (peak C)**	Ip (CH+CCC) bending
**1380**	Ring torsion<br>CH2 wagging
**1417-1422 (D peak)**	Ip (CH+CCC) bending<br>CC stretching
**1437-1443 (B peak)**	Ip (CH+CCC) bending<br>CH deformation<br>CC stretching
**1506-1537 (A peak)**	Ip (CH+CCC) bending<br>CCC asymmetrical deformation<br>CC stretching
**1590 (E peak)**	Ip (CH+CCC) bending<br>CC stretching
**1615 (F peak)**	Ip (CH+CCC) bending<br>CC ring stretching

where ip is in-plane and oop is out-of-plane.

Topography by atomic force microscopy

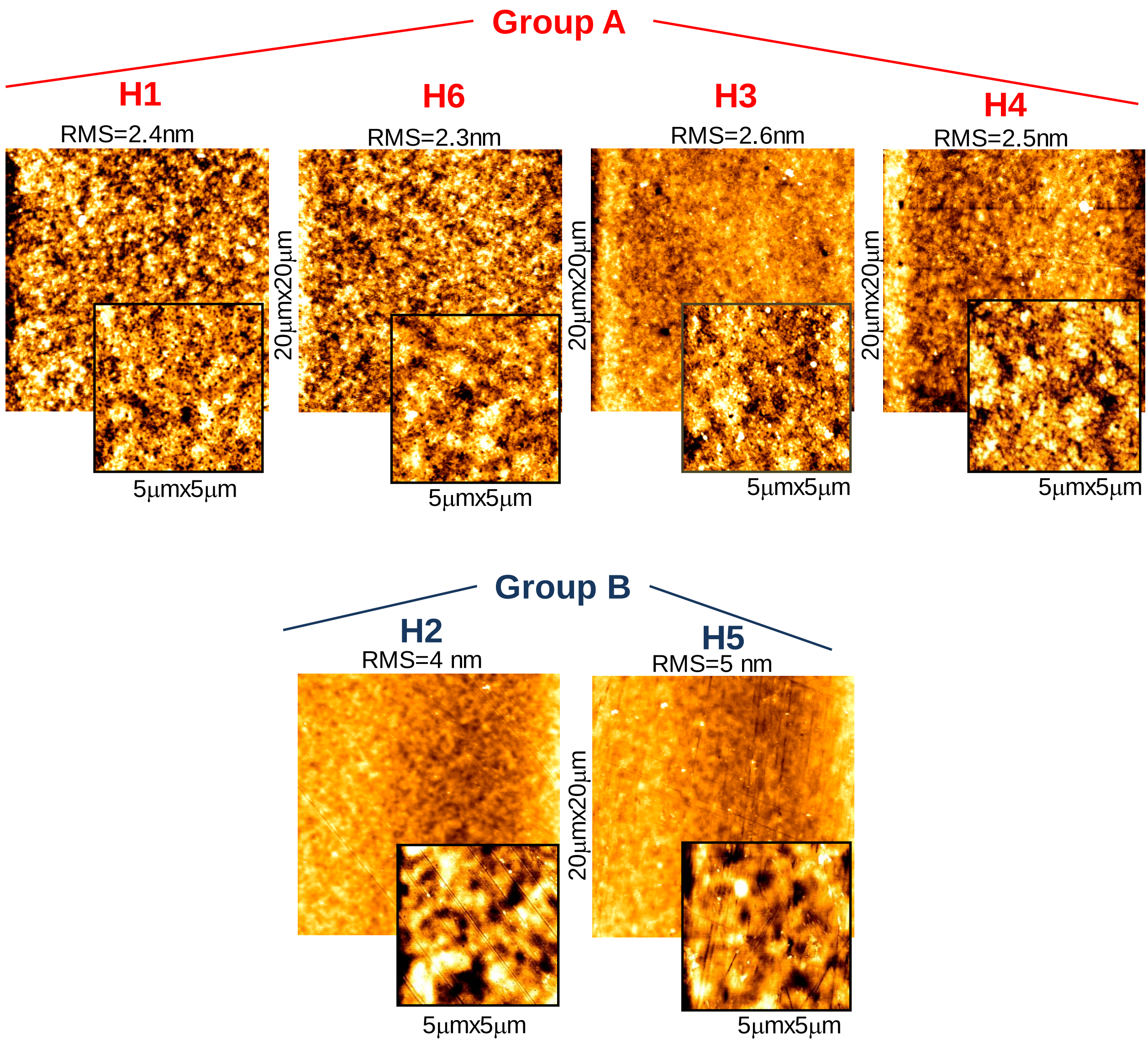

Figure S5: 20 µmx20 µm topographical images, with corresponding zoomed 5 µmx5 µm ones, of **H1**, **H2**, **H3**, **H4**, **H5** and **H6** samples grouped in Group A and Group B.

Nuclear magnetic resonance spectra

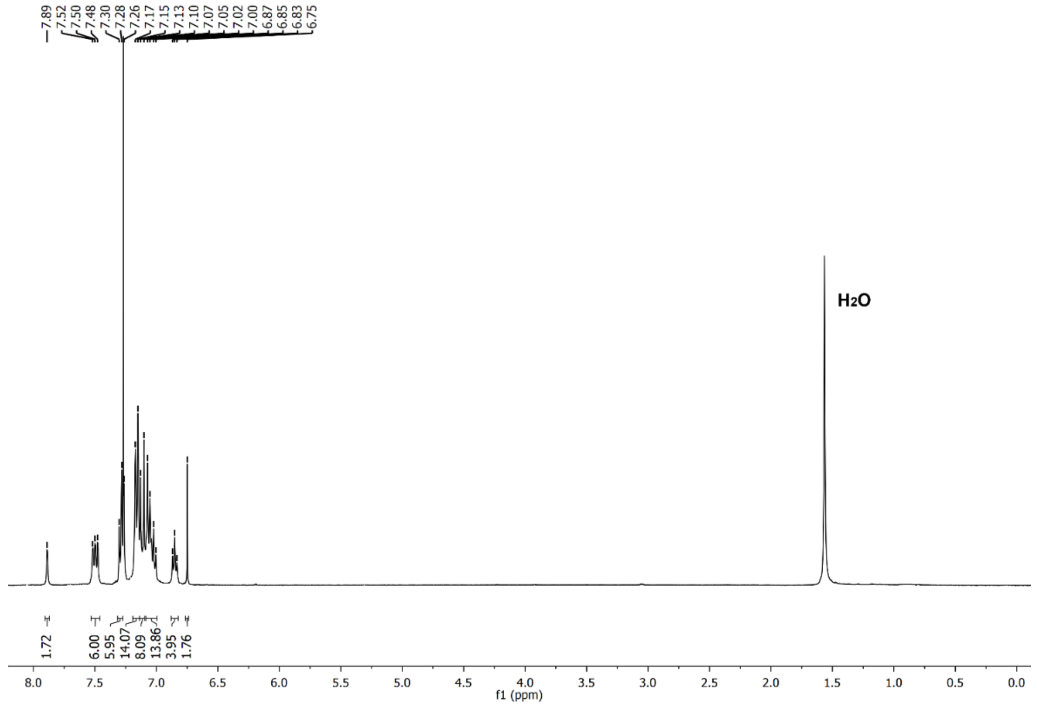

Figure S6: ^{1}H NMR (400 MHz, $CDCl_3$) of **H3.**

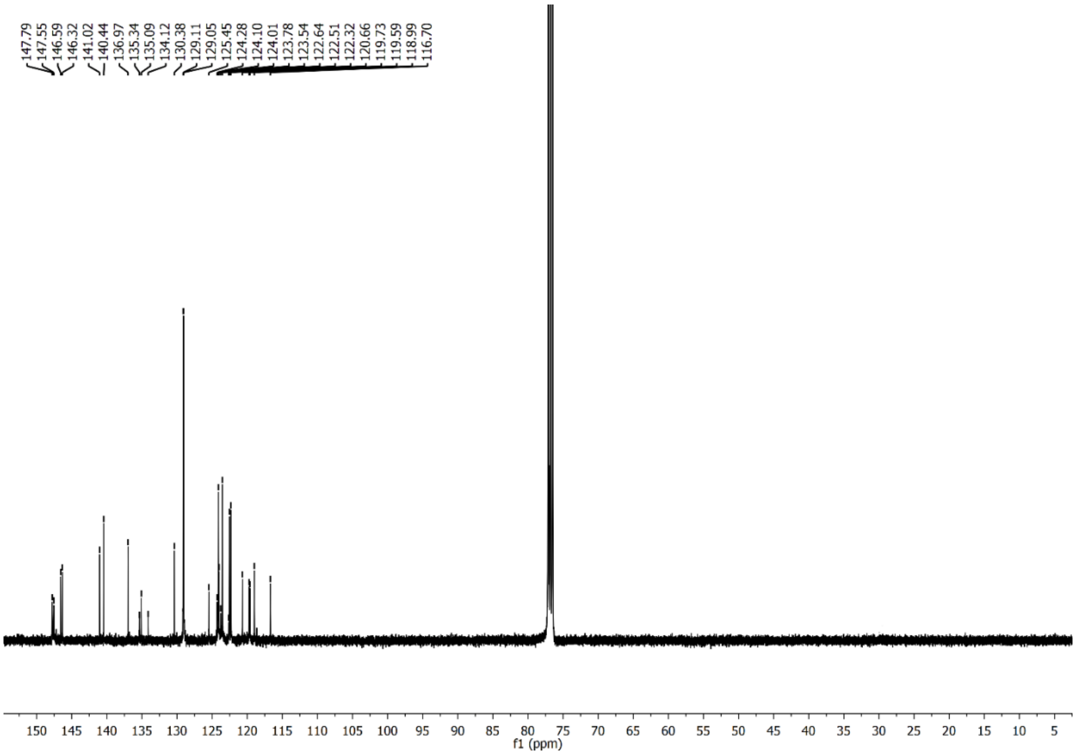

Figure S7: ^{13}C NMR (101 MHz, CDCl3) of **H3.**

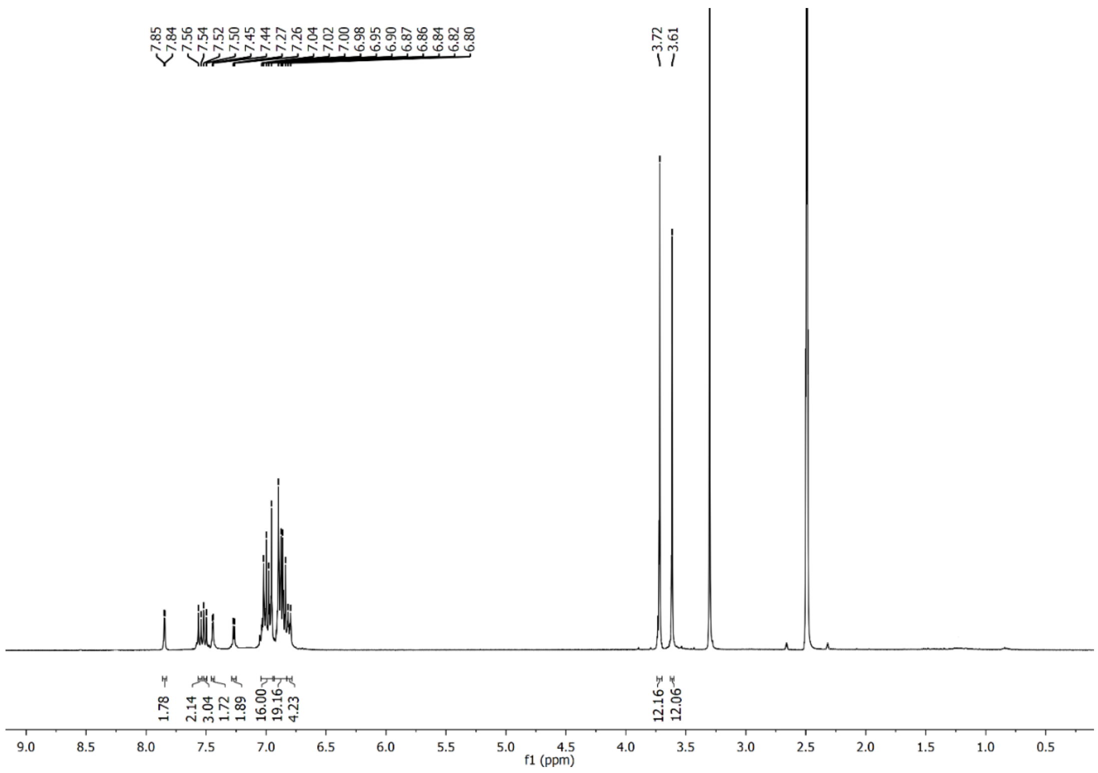

Figure S8: ^{1}H NMR (400 MHz, DMSO-d_6) of **H4.**

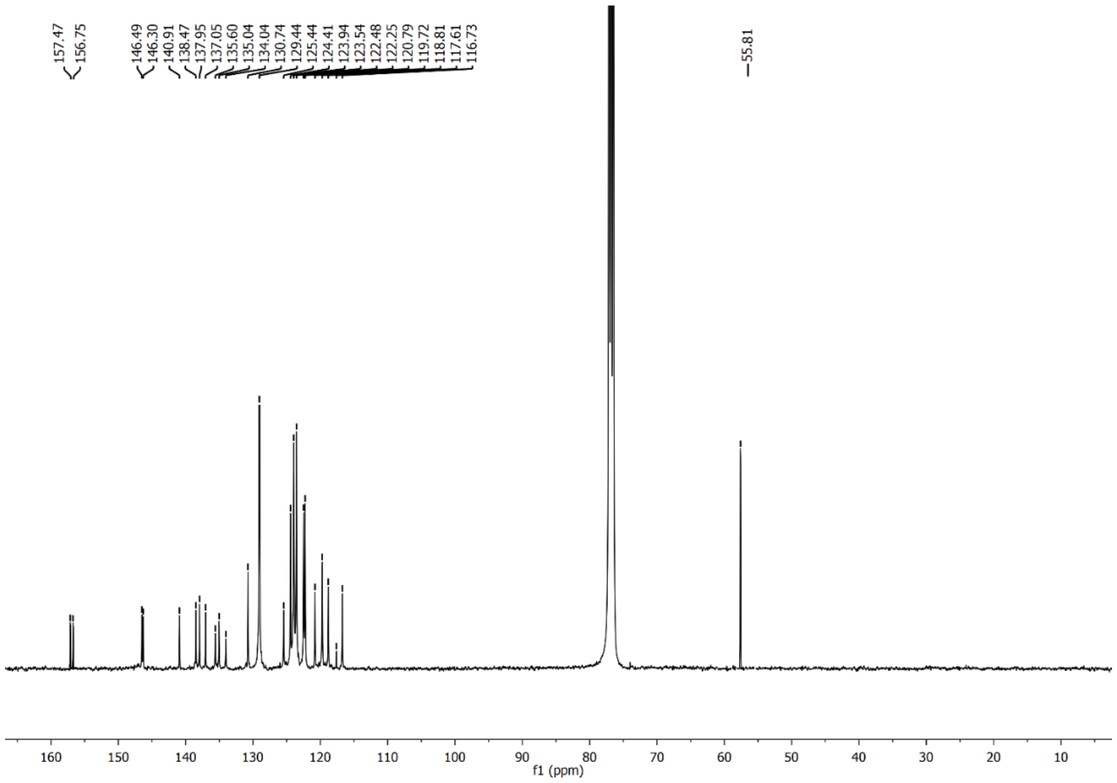

Figure S9: ^{13}C NMR (101 MHz, CDCl3) of **H4.**

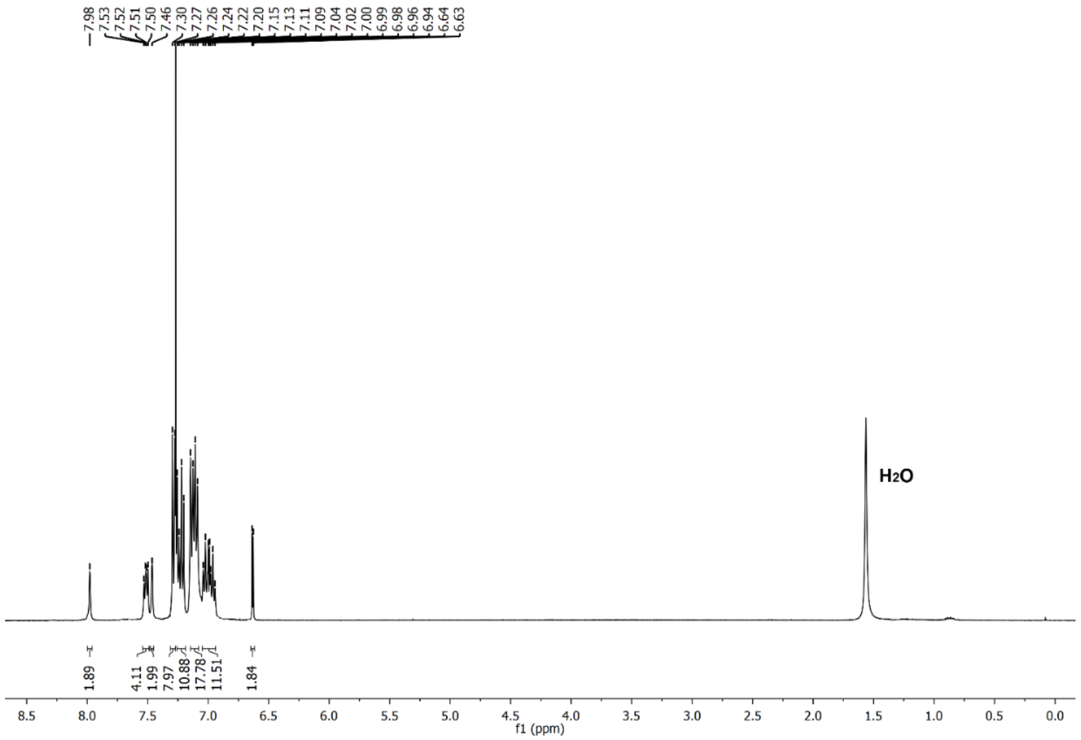

Figure S10: ^{1}H NMR (400 MHz, $CDCl_3$) of **H6.**

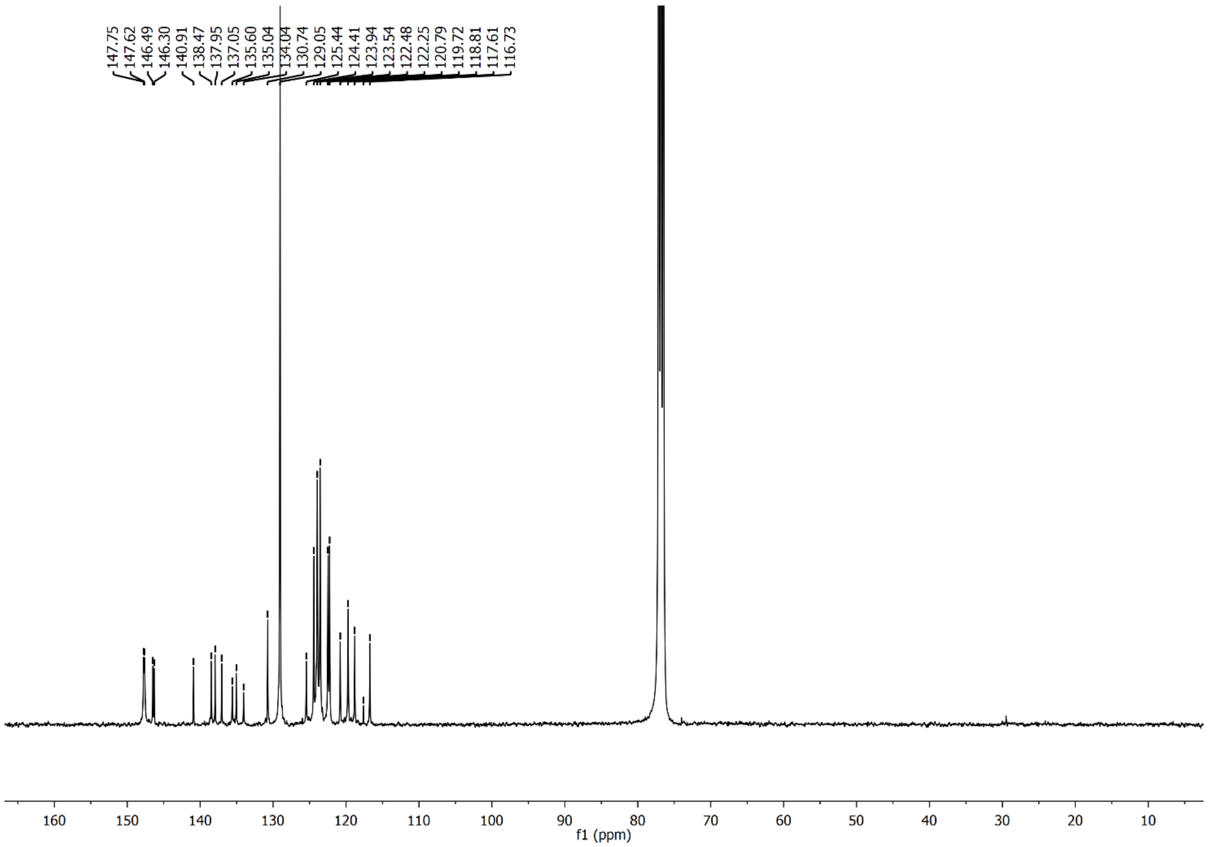

Figure S11: 13C NMR (101 MHz, CDCl3) of **H3.**

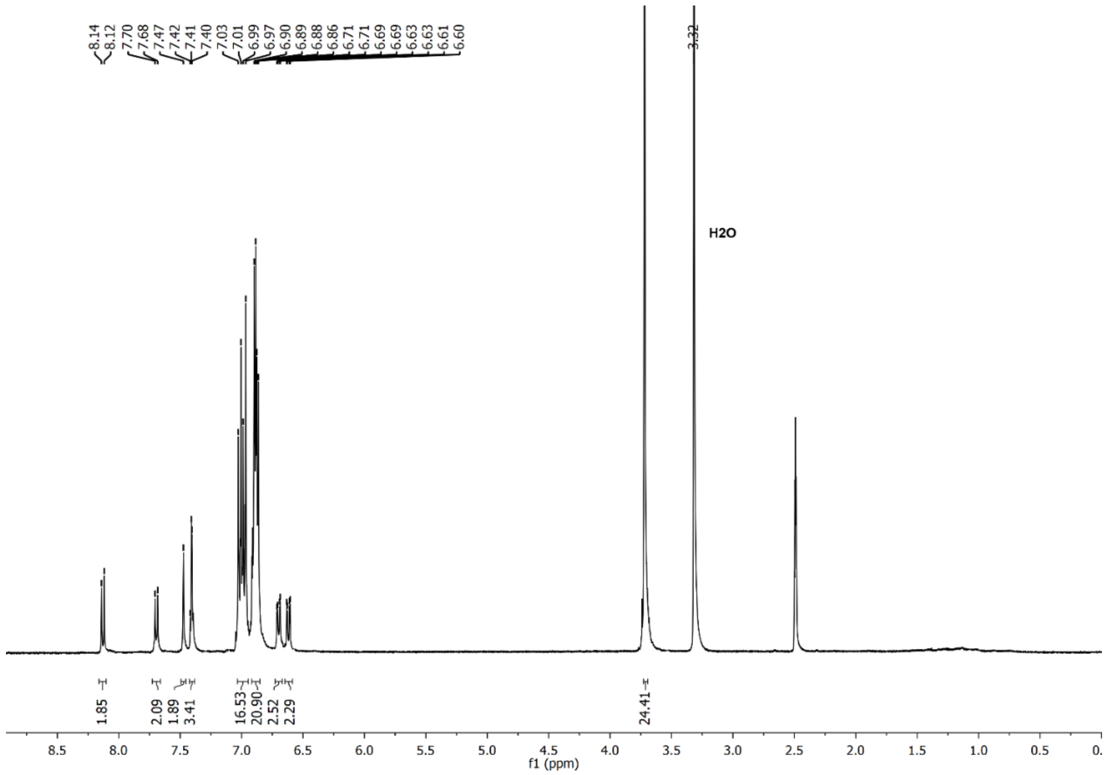

Figure S12: ^{1}H NMR (400 MHz, DMSO-d_6) of **H5.**

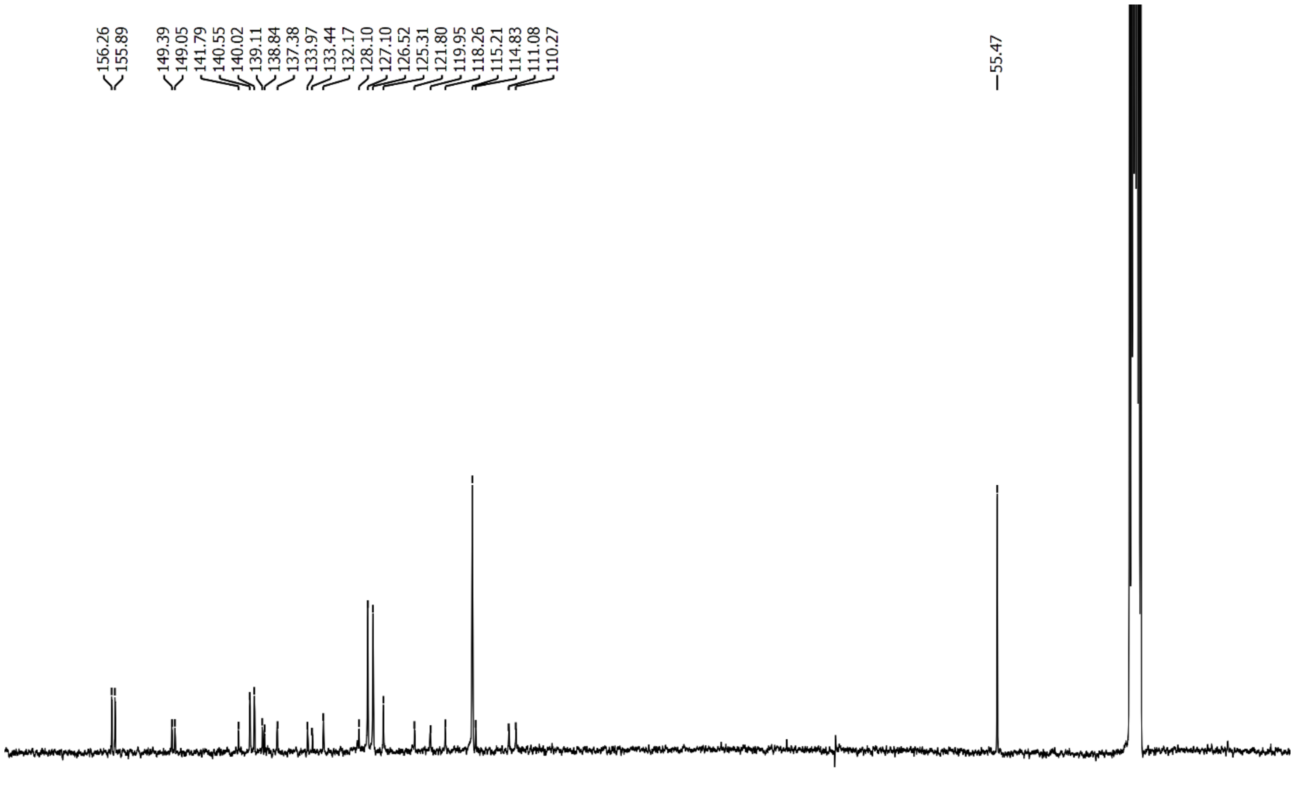

Figure S13: ^{13}C NMR (101MHz, DMSO-d_6) of **H5.**
